# Longitudinal Plasma Metabolomics Profile in Pregnancy—A Study in an Ethnically Diverse U.S. Pregnancy Cohort

**DOI:** 10.3390/nu13093080

**Published:** 2021-09-01

**Authors:** Susanna D. Mitro, Jing Wu, Mohammad L. Rahman, Yaqi Cao, Yeyi Zhu, Zhen Chen, Liwei Chen, Mengying Li, Stefanie N. Hinkle, Andrew A. Bremer, Natalie L. Weir, Michael Y. Tsai, Yiqing Song, Katherine L. Grantz, Bizu Gelaye, Cuilin Zhang

**Affiliations:** 1 Epidemiology Branch, Division of Intramural Population Health Research, Eunice Kennedy Shriver National Institute of Child Health and Human Development, National Institutes of Health, Bethesda, MD 20817, USA; susanna.mitro@nih.gov (S.D.M.); mengying.li@nih.gov (M.L.); stefanie.hinkle@pennmedicine.upenn.edu (S.N.H.); katherine.grantz@nih.gov (K.L.G.); 2 Glotech, Inc., Rockville, MD 20850, USA; jing.wu2@nih.gov; 3 Department of Population Medicine and Harvard Pilgrim Health Care Institute, Harvard Medical School, Boston, MA 02115, USA; mohammad.rahman2@nih.gov; 4 Department of Biostatistics, Epidemiology and Informatics, University of Pennsylvania Perelman School of Medicine, Philadelphia, PA 19104, USA; yaqi.cao@pennmedicine.upenn.edu; 5 Division of Research, Kaiser Permanente Northern California, Oakland, CA 94612, USA; yeyi.zhu@kp.org; 6 Biostatistics and Bioinformatics Branch, Division of Intramural Population Health Research, Eunice Kennedy Shriver National Institute of Child Health and Human Development, National Institutes of Health, Bethesda, MD 20817, USA; chenzhe@mail.nih.gov; 7 Department of Epidemiology, Fielding School of Public Health, University of California Los Angeles, Los Angeles, CA 90095, USA; cliwei86@g.ucla.edu; 8 Pediatric Growth and Nutrition Branch, Eunice Kennedy Shriver National Institute of Child Health and Human Development, National Institutes of Health, Bethesda, MD 20817, USA; Andrew.bremer@nih.gov; 9 Department of Laboratory Medicine and Pathology, University of Minnesota, Minneapolis, MN 55455, USA; weirx065@umn.edu (N.L.W.); tsaix001@umn.edu (M.Y.T.); 10 Department of Epidemiology, Indiana University Richard M. Fairbanks School of Public Health, Indianapolis, IN 46202, USA; yiqsong@iu.edu; 11 Department of Epidemiology, Harvard T.H. Chan School of Public Health, Boston, MA 02115, USA; bgelaye@hsph.harvard.edu

**Keywords:** pregnancy, targeted metabolomics, longitudinal, non-linear, fatty acids, amino acids, acylcarnitines

## Abstract

Amino acids, fatty acids, and acylcarnitine metabolites play a pivotal role in maternal and fetal health, but profiles of these metabolites over pregnancy are not completely established. We described longitudinal trajectories of targeted amino acids, fatty acids, and acylcarnitines in pregnancy. We quantified 102 metabolites and combinations (37 fatty acids, 37 amino acids, and 28 acylcarnitines) in plasma samples from pregnant women in the *Eunice Kennedy Shriver* National Institute of Child Health and Human Development (NICHD) Fetal Growth Studies—Singletons cohort (*n* = 214 women at 10–14 and 15–26 weeks, 107 at 26–31 weeks, and 103 at 33–39 weeks). We used linear mixed models to estimate metabolite trajectories and examined variation by body mass index (BMI), race/ethnicity, and fetal sex. After excluding largely undetected metabolites, we analyzed 77 metabolites and combinations. Levels of 13 of 15 acylcarnitines, 7 of 25 amino acids, and 18 of 37 fatty acids significantly declined over gestation, while 8 of 25 amino acids and 10 of 37 fatty acids significantly increased. Several trajectories appeared to differ by BMI, race/ethnicity, and fetal sex although no tests for interactions remained significant after multiple testing correction. Future studies merit longitudinal measurements to capture metabolite changes in pregnancy, and larger samples to examine modifying effects of maternal and fetal characteristics.

## 1. Introduction

Metabolomics, the study of small molecules in plasma or other biological compartments, can describe a person’s complex metabolic state at the time of measurement. Because pregnancy is a time of rapid metabolic change, and the etiology of many pregnancy complications is incompletely understood, metabolomics has recently emerged as a promising tool to examine the role of nutrients and metabolites in the etiology of maternal and neonatal complications [1]. For example, alterations in amino acids, fatty acids, and acylcarnitines have been significantly associated with gestational diabetes [2,3,4,5,6], and concentrations of acylcarnitines and amino acids have been linked to small for gestational age [7]. Because of these links between metabolites and maternal and fetal outcomes, describing longitudinal plasma concentrations of these metabolites across pregnancy is critical.

However, continuous longitudinal changes of multiple classes of plasma metabolites over pregnancy have not been described. Three recent studies described longitudinal changes in targeted maternal plasma metabolomics (including amino acids, fatty acids, and acylcarnitines) over healthy pregnancy, but each collected samples at the same fixed times in all participants, leaving gaps in the remaining weeks of pregnancy and precluding description of continuous changes over time [8,9,10]. A few additional preliminary studies have measured metabolites (amino acids, fatty acids, and others) in maternal plasma at multiple points in pregnancy, but were all limited by methodological challenges including a small sample size (30 or fewer participants at each time point) and lack of repeated measurements on the same women [11,12,13,14].

Additionally, changes in metabolite concentration may be affected by maternal and fetal factors. For example, a number of studies (nearly all cross-sectional) have shown that maternal plasma metabolites (non-esterified fatty acids, branched chain amino acids, and some acylcarnitines, among others) differ by pre-pregnancy body mass index (BMI) [9,15,16,17,18,19]. Fetal sex may affect metabolites in maternal plasma or amniotic fluid [20,21], and one study in healthy pregnancy reported subtle differences in metabolomic profile by Hispanic ethnicity [8]. No study to our knowledge has explored how longitudinal changes in metabolites may differ by maternal BMI, fetal sex, and maternal race/ethnicity.

In this study, we used a multi-ethnic longitudinal pregnancy cohort with repeated maternal plasma samples to examine trajectories of targeted metabolites (amino acids, acylcarnitines, and phospholipid fatty acids) over the course of pregnancy among low-risk women without major pre-existing conditions. We additionally investigated variations of metabolite trajectories by pre-pregnancy BMI, fetal sex, and race/ethnicity, and grouped metabolites by trajectory. The purpose of this research is to describe natural changes in targeted metabolite concentrations across pregnancy, which is a period of major metabolic shifts.

## 2. Materials and Methods

The *Eunice Kennedy Shriver* National Institute of Child Health and Human Development Fetal Growth Studies—Singletons cohort was a multiracial prospective pregnancy cohort of 2802 women (2009–2013). Eligible women, recruited at 12 clinical sites, were 18–40 years old, had a singleton pregnancy <13 weeks without fetal congenital structural or chromosomal anomalies, and had a pre-pregnancy BMI between 19.0 and 45.0 kg/m^2^. Women with certain preexisting medical conditions or previous pregnancy complications were excluded. The analytic sample used for this analysis was a subset of the whole study population, restricted to women with quantified metabolites (*n* = 214 at visits 1 and 2, *n* = 107 at visit 3, and *n* = 103 at visit 4). Additional details on selection of participants into the analytic sample can be found in Supplemental Figure S1. Institutional Review Board approval was obtained for all participating clinical sites, the data coordinating center, and NICHD (May 2009, IRB number: 09-CH-N152). The research conforms to the principles of the Declaration of Helsinki. Written informed consent was obtained from all participants. This study was registered at www.clinicaltrials.gov (assessed on 30 August 2021) as NCT00912132. A detailed description of the cohort has been published [22].

### 2.1. Quantification of Metabolites

Blood samples were collected from study participants at four study visits during pregnancy, at 10–14 weeks (baseline visit), 15–26 weeks, 23–31 weeks, and 33–39 weeks (staggered to ensure that each week of pregnancy would be adequately sampled). At the first post-baseline visit, participants gave blood samples after an overnight fast of 8–14 h; at the baseline visit, second post-baseline visit, and last visit participants gave non-fasting samples. Investigators processed all blood samples immediately and stored the plasma samples at −80 °C until biomarker analysis, a method which has been shown to preserve metabolite stability [23].

Metabolites were measured in maternal plasma samples in a previous gestational diabetes case-control study nested in the NICHD Fetal Growth Studies—Singletons cohort. The case-control study identified 107 women who developed gestational diabetes, and individually matched each case to two controls by age (±2 years), race/ethnicity (Non-Hispanic White, Non-Hispanic Black, Hispanic, Asian/Pacific Islander), and gestational week of study visit (±2 weeks). Metabolites were measured in all gestational diabetes cases and matched controls for two study visits occurring before gestational diabetes diagnosis, and in all cases and one of the two matched controls for two study visits after diagnosis. For this study, we used metabolomics measurements from the controls (*n* = 214 at visits 1 and 2, *n* = 107 at visit 3, and *n* = 103 at visit 4) who are more representative of pregnant women in general than gestational diabetes cases (Supplemental Figure S1).

We quantified 93 targeted metabolites (28 acylcarnitines, 36 amino acids, and 29 phospholipid fatty acids). We measured acylcarnitines using electrospray ionization tandem mass spectrometry (ESI-MS/MS) [24]. Briefly, six internal standards of known concentration were added to the plasma sample, and samples were deproteinized with acetonitrile. Following shaking and centrifugation, the supernatants were dried and derivatized with n-butanolic HCl, yielding acylcarnitines in n-butyl-ester form. Samples were resuspended in running buffer and analyzed by ESI-MS/MS. The concentrations of the analytes were estimated by computerized comparison to internal standards. We measured phospholipid fatty acid profiles using EDTA plasma using a method previously described [25]. Briefly, lipids were extracted with chloroform/methanol, separated using thin-layer chromatography, and the phospholipid band was derivatized to methyl esters. The final product was injected onto a capillary Varian CP7420 100-m column with a Hewlet Packard 5890 gas chromatograph with a flame ionization detector interphased with HP Chemstation software. Fatty acids are expressed as a percent of total phospholipid fatty acids. Finally, we measured amino acids on an Amino Acid analyzer (Hitachi L-8900). Samples were deproteinized, acidified, and injected into a high-performance liquid chromatography system. The amino acids were eluted based on the pKa using cation exchange resin and multiple buffer and temperature gradients to resolve compounds. The column effluent was reacted at high temperature with ninhydrin and the wavelength absorbance was monitored. Each amino acid was quantified relative to standards of known concentrations. Names of fatty acids are structured as C(number of carbons):(number of double bonds)n(location of first double bond), followed by c and/or t to denote cis or trans bonds.

### 2.2. Covariate Measurement

At baseline, participants reported demographic characteristics including race/ethnicity, education, household income, marital status, and parity. Research staff recorded fetal sex from the neonatal discharge summary. We calculated pre-pregnancy BMI (weight (kg)/height (m)^2^) using self-reported pre-pregnancy weight and height measured by research staff at enrollment. At each blood draw, participants also reported the last time they had anything to eat or drink. For this descriptive analysis, we chose *a priori* to estimate metabolite trajectories by strata of maternal BMI (<25, 25–29.9, ≥30 kg/m^2^), fetal sex (male, female), and maternal race/ethnicity (Non-Hispanic White, Non-Hispanic Black, Hispanic, Asian/Pacific Islander).

### 2.3. Statistical Analysis

We excluded from analysis metabolites missing or undetected in more than 20% of measurements (*n* = 10 metabolites) and metabolites with a concentration of 0 in more than 50% of samples (*n* = 15 metabolites). For other metabolites with values below the detection limit (*n* = 11 acylcarnitines), we imputed values below the detection limit with the minimum/2 [26]. After exclusions, 68 metabolites remained: 15 acylcarnitines, 24 amino acids, and 29 phospholipid fatty acids. We additionally calculated 9 combinations (sums and ratios) of metabolites, for a total of 77 metabolites and combinations used in analysis. A list of quantified metabolites and ratios of metabolites can be found in the Supplemental Material Table S1.

Because the analytic sample is composed of the matched controls from a nested case-control study, we weighted the participants in our analytic sample at each visit by the inverse of the probability of selection, to return the distribution of demographic characteristics in the sample to baseline distributions for the cohort [27].

### 2.4. Linear Trajectories

First, we evaluated whether metabolite concentrations changed significantly over pregnancy using linear mixed effects models with random slopes and intercepts for time (modeled continuously; slopes with *p* < 0.05 were considered to significantly change). We repeated these analyses including main effects and product terms for pre-pregnancy BMI, fetal sex, and race/ethnicity, to test whether metabolite trajectories significantly differed by these characteristics. Models were not adjusted for other demographic covariates in the main analysis. When the estimated G-matrix was not positive definite for the overall or stratified analyses (*n* = 20 metabolites), we estimated metabolite trajectories using linear mixed effects models with random intercepts and fixed slopes for time. We corrected all sets of analyses for multiple testing using Benjamini-Hochberg false discovery rate correction [28]. We considered corrected *p*-values < 0.05 to be statistically significant.

### 2.5. Flexible Non-Linear Trajectories

Next, we relaxed the assumption of linear change in metabolite levels over time to model metabolite trajectories more precisely. We estimated flexible metabolite trajectories using mixed effects models with random slopes and intercepts for time, using penalized linear splines with knots every 5 gestational weeks (at 15, 20, 25, 30, and 35 weeks), not adjusted for demographic covariates. As in the linear models, when the estimated G-matrix was not positive definite (*n* = 20 metabolites), we estimated metabolite trajectories using linear mixed effects models with random intercepts and fixed slopes for time and no penalized splines. We used these models to estimate predicted values for each metabolite for gestational weeks 10–39.5 by 0.5-week intervals [29]. We plotted the observed data and estimated penalized spline for each metabolite (Supplemental Material).

### 2.6. Grouping Flexible Trajectories

Finally, we grouped metabolite trajectories using a non-parametric partitioning algorithm, k-means for longitudinal data (KML) [30]. We grouped trajectories to identify metabolites with similar trajectories, to which may indicate broad metabolic changes affecting multiple pathways. We scaled the predicted values of each metabolite from the penalized spline models to be between 0 and 1 to put all metabolites on one scale. We tested 2–10 clusters of trajectories, with 10 re-drawings per tested cluster number. We used the Calinski & Harabatz criterion to select the optimal number of clusters [30]. Scaling the predicted values allows for comparison of trajectory shape across metabolites but removes information about absolute slope (because the minimum is fixed to 0 and maximum is fixed to 1 for each metabolite). Observed slopes from penalized spline models for each metabolite are plotted in the Supplemental Material. We conducted analyses except KML in SAS 9.4 (SAS Institute Inc., Cary, NC, USA), and conducted KML in R version 3.5.1.

### 2.7. Sensitivity Analyses

To evaluate the effect of the fasting visit on estimated trajectories, we ran linear models dropping the fasting visit. To evaluate the robustness of models testing interaction by BMI, fetal sex, and race/ethnicity, we additionally adjusted them by the other factors (e.g., models testing fetal sex were adjusted for BMI and race/ethnicity). Finally, we used bootstrapping with 200 replicates to confirm the standard error of our weighted analyses; the bootstrapped confidence intervals were similar to those estimated from weighted linear mixed models.

## 3. Results

Participants ranged in age from 18–40 years, and the population was racially diverse: 31% of women were non-Hispanic White, 24% were non-Hispanic Black, 27% were Hispanic, and 18% were Asian/Pacific Islander (Table 1). About half of the women (52%) had pre-pregnancy BMIs <25 kg/m^2^, and 75% had at least some college education (Table 1).

### 3.1. Metabolite Concentrations over Time

**Figure 1:**
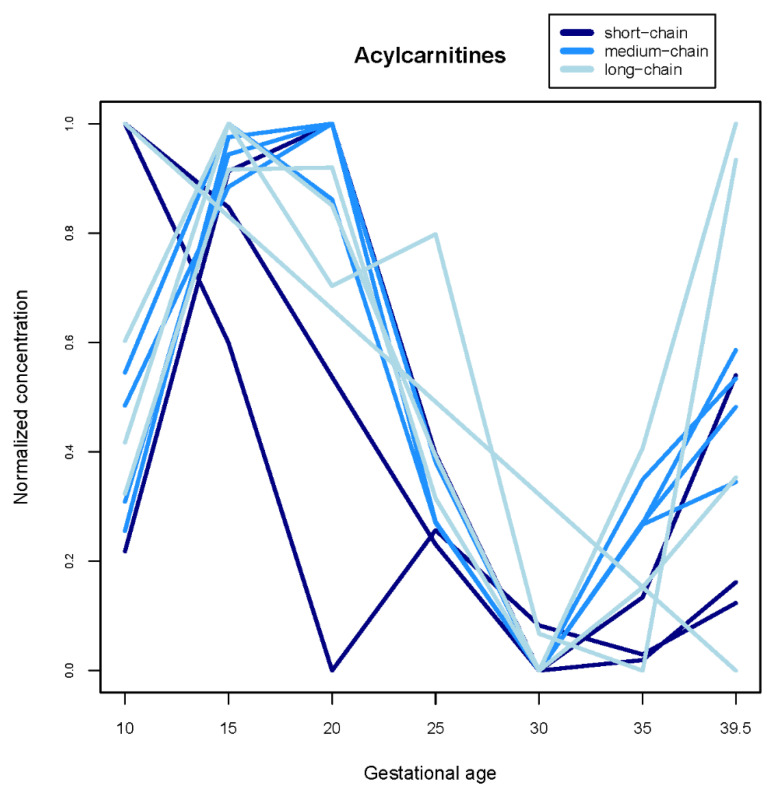
Trajectories of normalized acylcarnitine concentrations estimated from linear mixed models with 5-knot penalized splines. Short-chain acylcarnitines have 2–4 carbon chains, medium-chain acylcarnitines have 8–12 carbon chains, and long chain acylcarnitines have 14–18 carbon chains. Metabolites with the same trajectory are plotted as a single line. Absolute concentrations of each individual acylcarnitine (*n* = 15) are plotted in the Supplemental Material.

Concentrations of most acylcarnitines (13 of 15) significantly declined over gestation (Table 2), and changes appeared somewhat nonlinear when we flexibly modeled trajectories (Figure 1 and Supplemental Material).

**Figure 2:**
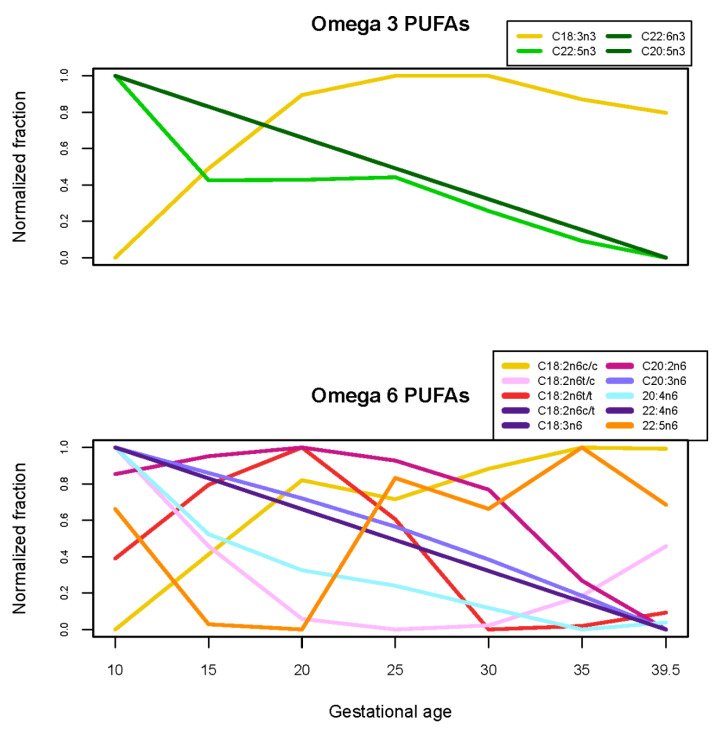
Trajectories of normalized relative percentages of omega-3 and omega-6 phospholipid polyunsaturated fatty acids (PUFAs), estimated from linear mixed models with 5-knot penalized splines. Metabolites with the same trajectory are plotted as a single line. Relative percentages of each phospholipid fatty acid (*n* = 37, including phospholipid fatty acids that are not omega-3 or omega-6) are plotted in the Supplemental Material.

**Figure 3:**
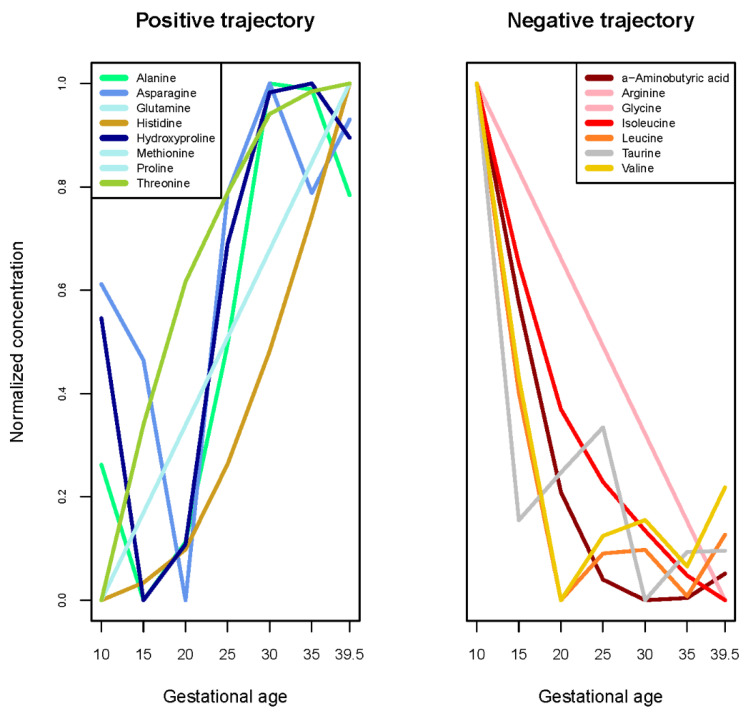
Trajectories of normalized amino acid concentrations, estimated from linear mixed models with 5-knot penalized splines. Amino acids with positive and negative trajectories were identified using slopes from linear mixed models. Metabolites with the same trajectory are plotted with the same color as a single line. Absolute concentrations of each amino acid (*n* = 25) are plotted in the Supplemental Material.

Among 37 tested fatty acids and sums, relative percentages of 8 fatty acids and 2 sums (Σ(cis fatty acids) and Σ(18:1 cis fatty acid isomers)) significantly increased, relative percentages of 14 fatty acids and 4 sums and ratios (Σ[trans fatty acids], eicosapentaenoic acid [EPA]/docosahexaneonic acid [DHA], arachindonic acid [AA]/DHA, AA/Σ[EPA,DHA]) significantly decreased, and relative percentages of the remaining 7 fatty acids and 2 ratios (AA/EPA, Σ[18:1 trans fatty acid isomers]) did not significantly change (Table 2). Relative percentages of essential omega-3 and omega-6 fatty acids (C18:3n3 and C18:2n6c/c, respectively) both increased, though trajectories for other omega-3 and omega-6 fatty acids varied (Table 2, Figure 2). Finally, of the 25 tested amino acids, concentrations of 8 significantly increased, concentrations of 7 significantly decreased, and the remaining 10 amino acids and 1 sum (Σ[Glutamic acid, glutamine]) did not significantly change (Table 2, Figure 3). Among the amino acids that did not significantly change, several (e.g., phenylalanine, ornithine, tyrosine) appear to have non-linear trajectories (Supplemental Material).

### 3.2. Trajectory Variation by BMI, Fetal Sex, Race/Ethnicity

Trajectories of 5 fatty acids and ratios (C16:0, C16:1n7c, C17:0, C20:3n6, [AA/Σ(EPA+DHA)]), dodecenoylcarnitine, and alanine differed by maternal BMI. Compared to trajectories among women with normal BMI, relative percentages of C16:1n7c and C20:3n6 declined significantly more, while relative percentage of AA/Σ(EPA + DHA) declined significantly less, among women with obese BMI. Relative percentages of C17:0 also declined significantly less in pregnancies of women with overweight or obese pre-pregnancy BMI compared to women with normal BMI. Concentrations of dodecenoylcarnitine declined significantly more and relative percentages of C16:0 increased significantly less in pregnancies of women with overweight BMI, compared to normal BMI. None of these interactions remained statistically significant after FDR correction (Supplemental Table S2).

Trajectories of 2 fatty acids (C15:0, C22:0) and 2 acylcarnitines (stearoylcarnitine, propionylcarnitine) significantly differed by fetal sex. Relative percentages of C15:0 and C22:0, and concentrations of stearoylcarnitine, and propionylcarnitine, declined more or increased less in pregnancies with male compared to female fetuses. However, these interactions ceased to be statistically significant after FDR correction (Supplemental Table S2).

Trajectories of six fatty acids (C18:1n7c, C18:3n6, C20:2n6, C20:3n6, C20:4n6, C22:0) and proline significantly differed by maternal race/ethnicity. Compared to non-Hispanic white women, relative percentages of C18:1n7c, C20:2n6, and C20:4n6 declined significantly less, and relative percentages of C18:3n6 and C20:3n6 declined significantly more, in pregnancies of non-Hispanic Black women; relative percentages of C22:0 rose significantly less, while concentrations of proline rose significantly more, in pregnancies of non-Hispanic Black, Hispanic, and Asian/Pacific Islander women. Again, no interactions were statistically significant after FDR correction (Supplemental Table S2). Supplemental Table S3 shows all instances where either slope or intercept significantly varied by race/ethnicity, fetal sex, or BMI.

### 3.3. Grouping Flexible Components

**Figure 4:**
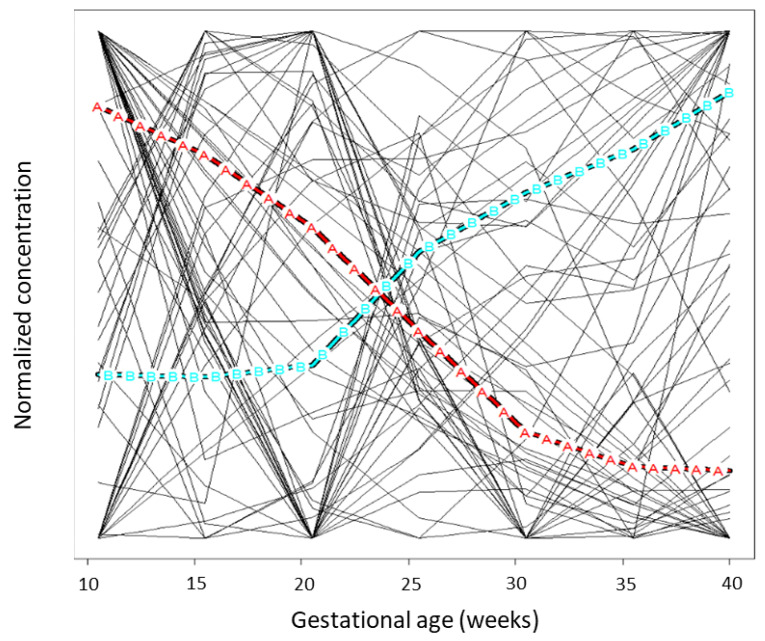
The two groups of trajectories (A, B) selected by the k-means for longitudinal data algorithm. The black lines behind the labeled trajectories represent the trajectories being grouped (1 trajectory per metabolite).

We grouped the flexible metabolite trajectories to identify metabolites with similar trajectories. Using the KML algorithm, the best fit was a model with 2 groups of trajectories (Figure 4). Group A, which is characterized by monotonic decreasing concentration, included all 15 acylcarnitines; 9 amino acids (α-aminobutyric acid, aspartic acid, isoleucine, leucine, taurine, valine, arginine, glutamic acid, glycine); and 25 fatty acids (C15:0, C17:0, C18:0, C18:1n6-9t, C18:1n7c, C18:1n6c, C18:2n6t/t, C18:2n6c/t, C18:2n6t/c, C18:3n6, C20:1n9, C20:2n6, C20:3n6, C20:4n6, C20:5n3, C22:4n6, C24:0, C22:5n3, C22:6n3, Σ[trans fatty acids], Σ[18:2 trans fatty acids], Σ[EPA, DHA], AA/EPA, AA/DHA, AA/Σ[EPA, DHA]). Group B, characterized by a relatively flat trajectory in gestational weeks 10–15 and then a positive trajectory through the end of gestation, consisted of the other 28 metabolites: 16 amino acids (alanine, asparagine, citrulline, cystine, glutamine, Σ[glutamic acid, glutamine], histidine, hydroxyproline, lysine, ornithine, phenylalanine, serine, threonine, tyrosine, methionine, proline) and 12 fatty acids (C14:0, C16:0, C16:1n7c, C18:1n9c, C18:2n6c/c, C18:3n3, C20:0, C22:0, C22:5n6, C24:1n9, Σ[cis fatty acids], Σ[18:1 cis fatty acids]). Group memberships are listed by metabolite class in Supplemental Table S1.

### 3.4. Sensitivity Analyses

Linear models without the fasting visit were similar to those including all data (Supplemental Table S4). Interaction models were sometimes slightly attenuated but generally robust to mutual adjustment by pre-pregnancy BMI, fetal sex, and race/ethnicity (not shown).

## 4. Discussion

In this longitudinal pregnancy cohort with four repeated measurements of three targeted metabolomics panels of 77 metabolites and combinations, including amino acids, phospholipid fatty acids, and acylcarnitines, we observed profound changes in metabolite concentrations in all classes. In addition, we observed suggestive evidence that trajectories of two saturated fatty acids and two acylcarnitines differed by fetal sex; five fatty acids and proline differed by maternal race/ethnicity; and five fatty acids, dodecenoylcarnitine, and alanine differed by pre-pregnancy BMI, though interactions were not significant after FDR correction. To our knowledge, this is the first longitudinal human epidemiological study to estimate continuous trajectories of targeted acylcarnitines, fatty acids, and amino acids over pregnancy and summarize flexible trajectories across classes.

Acylcarnitines play an essential role in fatty acid oxidation and accumulate in the fetus during gestation [31]. We reported that maternal concentrations of most acylcarnitines (except octenoylcarnitine and tetradecanoylcarnitine), significantly decreased during pregnancy, which largely aligns with patterns noted in prior work [8,9,12,32]. Interestingly, when we flexibly modeled acylcarnitine trajectories, we found that the significant decline we and others reported may be non-linear: many acylcarnitines appeared to decline in early/mid-pregnancy and rise somewhat after 30 weeks, though absolute changes remained small. It is not fully established why acylcarnitines decline in pregnancy, though increasing fetal metabolic demands may lead to greater transplacental transfer [33]. Alternatively, fatty acid oxidation may decrease as pregnancy progresses, producing fewer acylcarnitines as byproducts [8], or urinary excretion of acylcarnitines may increase [34].

Phospholipid fatty acids (especially long-chain polyunsaturated fatty acids) are increasingly transferred to the fetoplacental compartments in pregnancy [35]. Plasma fatty acids reflect both dietary intake and metabolic processes, and play many roles in pregnancy: they accumulate in the fetal nervous system [36], may alter maternal inflammation [37] and might affect development of gestational diabetes [3,4]. We did not find clear trajectory patterns by metabolite saturation. Previous findings are also inconsistent: one study that used absolute rather than relative concentrations of fatty acids reported no changes among saturated and monounsaturated fatty acids, while some long-chain polyunsaturated fatty acids decreased from first to second trimester [8]; another small study found that the proportion of long-chain and unsaturated fatty acids increased over pregnancy [13]. We found that relative percentages of both linoleic acid and alpha-linolenic acid increased over gestation, while relative percentages of DHA and AA declined and EPA did not significantly change. This was expected, as DHA and AA preferentially accumulate in the growing fetus [35].

Changes in amino acid concentrations may reflect adaptive transport of amino acids in response to fetal nutrition or endocrine signaling [38,39]. Maternal amino acid concentrations have been associated with intrauterine growth restriction [40] and gestational diabetes [41,42]. In alignment with prior studies, we found that concentrations of alanine [9,14], threonine [8,14], and glutamine [12] increased over pregnancy, while arginine, glycine, leucine, and valine significantly decreased over pregnancy [8,9,14]. Unlike earlier studies, we also reported decreases in isoleucine and increases in histidine, methionine, and proline [8,13]. Changes in multiple metabolites (e.g., isoleucine, histidine) appeared gradual or nonlinear, and studies using few measurements may not have been able to detect these subtle changes. As fetal energetic demands increase, maternal concentrations of most amino acids are expected to decline [14]. It is not clear why certain amino acids appeared to increase.

We estimated multiple non-linear metabolite trajectories and used KML to group the diverse trajectories, resulting in two groups (generally increasing and generally decreasing). It may be that these varied metabolites are best summarized by two groups, but this summary of the trajectories could also be an oversimplification. In order to group trajectories across classes of metabolites, we had to put all metabolites on the same scale; this may have magnified or distorted changes in slope that are subtle on the original scale. To study the metabolic complexity of pregnancy, future research should also explore simultaneous changes in metabolites across multiple classes, which might offer insight into shared biological pathways. Identification of metabolites with similar trajectories may assist in understanding the clinical implications of the timing and shape of metabolite concentration changes across pregnancy, and additional studies are warranted.

We observed suggestive evidence that trajectories of multiple metabolites may differ by BMI, fetal sex, and race/ethnicity. Before FDR correction, trajectories of five fatty acids and ratios, one acylcarnitine, and one amino acid differed by maternal BMI; two saturated fatty acids and two acylcarnitines differed significantly by fetal sex; and five fatty acids and one amino acid significantly differed by maternal race/ethnicity. Associations were not significant after FDR correction, and should be interpreted with caution. Future studies with larger sample size are warranted to confirm our findings.

Our study has several unique strengths. Our sample was diverse in race/ethnicity and pre-pregnancy BMI, which enabled us to investigate variability in metabolite trajectories across these factors. Additionally, our staggered repeated measurement design allowed us to smoothly model metabolite concentrations over pregnancy. We could also describe simultaneous changes in a variety of metabolites from multiple classes.

Our analysis also has potential limitations. Our moderate sample size limited power to test trajectories in subgroups, though our sample was among the largest in a longitudinal pregnancy metabolomics study. Our goal was to evaluate metabolite variation by sociodemographic factors in this analysis, but it is likely that metabolites also vary by other lifestyle and dietary factors, which warrants future examination. Variation in fasting status may have introduced variability in metabolite concentrations. However, we expect that variability to increase random noise, rather than artificially introduce trajectories. Also, phospholipid fatty acids and amino acids are not typically strongly affected by fasting status [23], and results were generally robust to exclusion of the fasting visit. The KML algorithm identified only two relatively simple trajectory groups, which did not lend themselves to easy clinical interpretation. This approach should be replicated in future research to further refine groups. Finally, we scaled the estimated metabolite trajectories to group them, which removes information about magnitude of changes on the original scale. However, we provide graphs of the true concentrations and trajectories in the Supplemental Material.

## 5. Conclusions

We examined trajectories of 77 metabolites and combinations of metabolites from three targeted panels including amino acids, phospholipid fatty acids, and acylcarnitines, and observed profound changes in all three classes over pregnancy. Future studies on these metabolites in association with pregnancy and neonatal outcomes merit consideration of longitudinal measurements instead of single point measurements.

## Figures and Tables

**Table 1:** Weighted demographic characteristics of study participants at enrollment (*n* = 214).

Characteristics	*n*^1^ (%)
**Maternal age (years)**	
18–24	65 (30.5)
25–29	64 (29.9)
30–34	55 (25.6)
35–40	30 (14.0)
**Race/ethnicity**	
Non-Hispanic White	67 (31.3)
Non-Hispanic Black	51 (23.7)
Hispanic	57 (26.8)
Asian/Pacific Islander	39 (18.3)
**Pre-pregnancy BMI (kg/m^2^)**	
<25	112 (52.4)
25–29.9	71 (33.0)
≥30	31 (14.6)
**Married or living with partner**	155 (72.3)
**Maternal education**	
Some high school	22 (10.4)
High school diploma	31 (14.5)
Some college	75 (34.8)
Bachelor’s degree	51 (23.9)
Graduate degree	35 (16.5)
**Annual household income**	
<$30,000	52 (24.3)
$30,000–$49,999	37 (17.1)
$50,000–$99,999	43 (20.0)
≥$100,000	50 (23.4)
Missing	32 (15.1)
**Infant Sex**	
Male	110 (51.3)
Female	103 (48.2)
Missing	1 (0.5)

^1^*n* correspond to weighted percentage.

**Table 2:** Summary of the direction of change for metabolites whose concentrations statistically significantly increased (positive trajectory) or decreased (negative trajectory) over pregnancy (after FDR correction), based on slopes from linear mixed effects models.

Class	Positive Trajectory	Negative Trajectory	No Significant Change
**Acylcarnitines**	--	Acetylcarnitine, Propionylcarnitine, Decenoylcarnitine, Decanoylcarnitine, Glutarylcarnitine, Dodecenoylcarnitine, Dodecanoylcarnitine, Tetradecenoylcarnitine, Hexadecenoylcarnitine, Hexadecanoylcarnitine, Linoleylcarnitine, Oleylcarnitine, Stearoylcarnitine	Octenoylcarnitine, Tetradecanoylcarnitine
**Fatty acids**	C16:0, C16:1n7c, C18:1n9c, C18:2n6c/c, C18:3n3, C22:0, C22:5n6, C24:1n9, Σ(C18:1 cis fatty acids), Σ(cis fatty acids)	C15:0, C17:0, C18:0, C18:1n6-9t, C18:1n6c, C18:1n7c, C18:2n6c/t, C18:2n6t/t, C20:1n9, C20:2n6, C20:4n6, C22:5n3, C22:6n3, C24:0, Σ(trans fatty acids), Σ(EPA, DHA), AA/DHA, AA/Σ(EPA, DHA)	C14:0, C18:2n6t/c, C18:3n6, C20:0, C20:3n6, C20:5n3, C22:4n6, AA/EPA, Σ(C18:2 trans fatty acids)
**Amino acids**	Alanine, Asparagine, Glutamine, Histidine, Hydroxyproline, Methionine, Proline, Threonine	α-aminobutyric acid, Arginine, Glycine, Isoleucine, Leucine, Taurine, Valine	Aspartic acid, Citrulline, Cystine, Glutamic acid, Σ(Glutamic acid, Glutamine), Lysine, Ornithine, Phenylalanine, Serine, Tyrosine

Abbreviations: AA—Arachidonic acid; EPA—Eicosapentaenoic acid; DHA—Docosahexaenoic acid. Fatty acids are formatted as C(number of carbons):(number of double bonds)n(location of first double bond). Where needed, fatty acid names are followed by c and/or t to denote cis or trans bonds.

## Data Availability

Data described in the manuscript, code book, and analytic code will be available upon request pending application and approval of a data sharing agreement.

## Supplementary Materials

The following are available online at https://www.mdpi.com/article/10.3390/nu13093080/s1, Figure S1: Participant flowchart, Table S1: Quantified targeted metabolites and ratios of metabolites measured in this cohort, Table S2: Linear trajectories for metabolites with significant interactions, Table S3: Linear trajectories for metabolites with significant differences in slope or intercept, Table S4: Linear models with all data and after excluding the fasting visit, Metabolite graphs.
