# Supplementary material for: Longitudinal Plasma Metabolomics Profile in Pregnancy—A Study in an Ethnically Diverse U.S. Pregnancy Cohort
Source: Nutrients. 2021 Sep 1;13(9):3080. doi: 10.3390/nu13093080 (PMC8471130; doi:10.3390/nu13093080)
Supplement: Supplementary file 1 [file nutrients-13-03080-s001.zip › nutrients-1340802-SI.pdf]

Supplemental Material

Longitudinal plasma metabolomics profile in pregnancy-- a study in an ethnically diverse U.S. pregnancy cohort

Susanna D. Mitro, Jing Wu, Mohammad L. Rahman, Yaqi Cao, Yeyi Zhu, Zhen Chen, Liwei Chen, Mengying Li, Stefanie N. Hinkle, Andrew A. Bremer, Natalie L. Weir, Michael Y. Tsai, Yiqing Song, Katherine L. Grantz, Bizu Gelaye, Cuilin Zhang

Supplemental Figure S1 ………..……………………………………………………………. p2

Supplemental Table S1………………………………………………………………………. p3

Supplemental Table S2……………………………………………………………..…………p7

Supplemental Table S3………………………………………………………………………. p8

Supplemental Table S4……………………………………………………………..……. p13

Metabolite graphs ……………………………………………………………………….…. p15

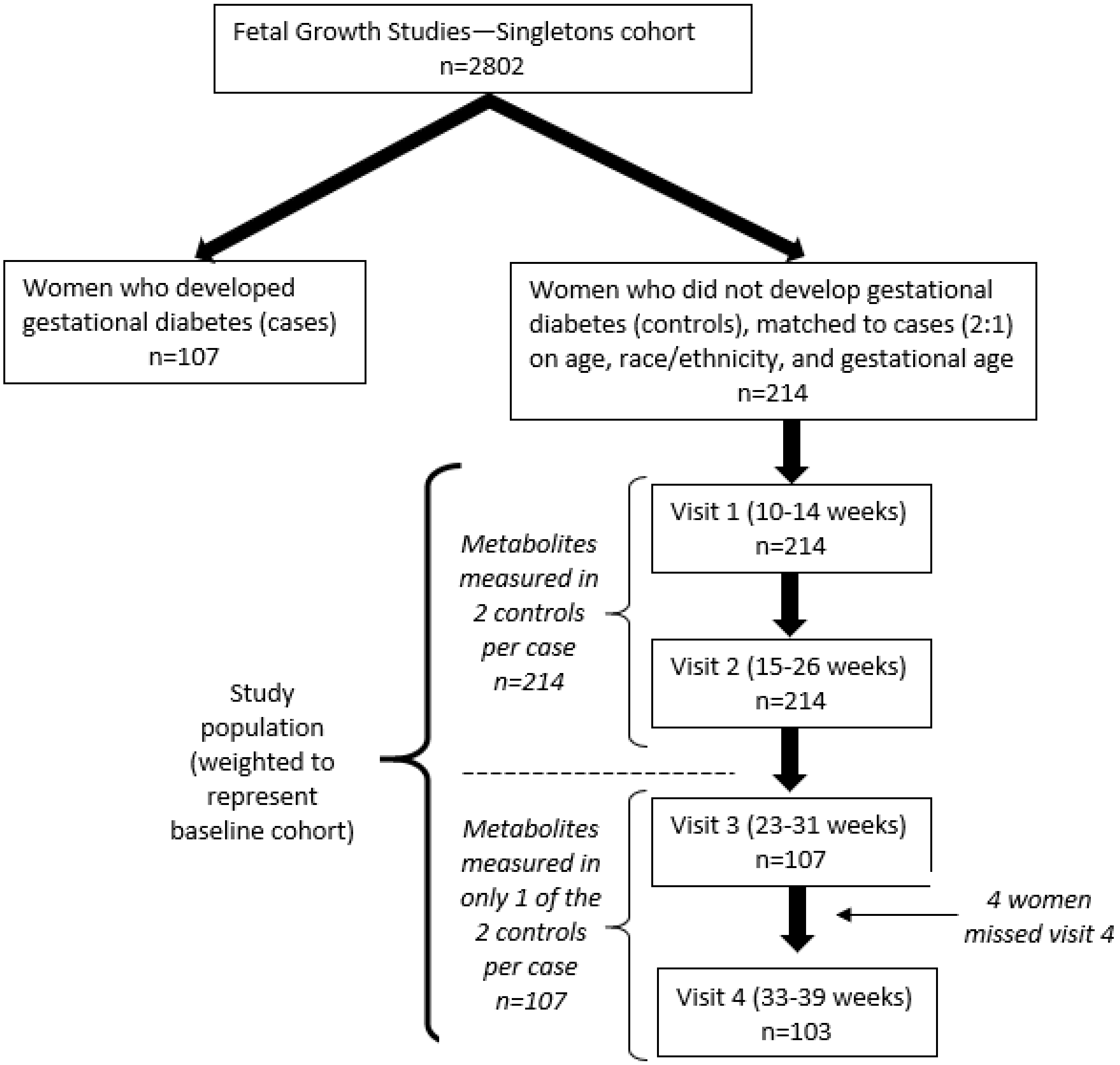

Supplemental Figure S1. Participant flowchart. The study population consists of the matched controls from a nested case-control study, weighted to represent the baseline cohort.

Supplemental Table S1: Quantified targeted metabolites and ratios of metabolites measured in this cohort. Metabolites excluded from analysis are noted with reason for exclusion. The direction of the linear change is based on slopes from linear mixed effects models; statistical significance is determined after FDR correction. KML groups correspond to those shown in Figure 4.

Class	Metabolite name	Included in analysis?	Significant linear change?	KML group
Acylcarnitines	Acetylcarnitine	Yes	Negative	A
	Propionylcarnitine	Yes	Negative	A
	Iso-/Butyrylcarnitine	No: Undetected >80%		
	Isovaleryl-/2-methylbutyrylcarnitine	No: Undetected >80%		
	Hexanoylcarnitine	No: Undetected >80%		
	3-OH-hexanoylcarnitine	No: Undetected >80%		
	Octenoylcarnitine	Yes	--	A
	Octanoylcarnitine	No: Undetected >80%		
	Decenoylcarnitine	Yes	Negative	A
	Decanoylcarnitine	Yes	Negative	A
	Glutarylcarnitine	Yes	Negative	A
	Dodecenoylcarnitine	Yes	Negative	A
	Dodecanoylcarnitine	Yes	Negative	A
	3-OH-dodecanoylcarnitine	No: Undetected >80%		
	Tetradecandienoylcarnitine	No: Undetected >80%		
	Tetradecenoylcarnitine	Yes	Negative	A
	Tetradecanoylcarnitine	Yes	--	A
	3-OH-tetradecenoylcarnitine	No: Undetected >80%		
	3-OH-tetradecanoylcarnitine	No: 0s in >50%		
	Hexadecenoylcarnitine	Yes	Negative	A
	Hexadecanoylcarnitine	Yes	Negative	A
	3-OH-hexadecenoylcarnitine	No: 0s in >50%		
	3-OH-hexadecanoylcarnitine	No: 0s in >50%		
	Linoleylcarnitine	Yes	Negative	A
	Oleylcarnitine	Yes	Negative	A
	Stearoylcarnitine	Yes	Negative	A

	3-OH-linoleylcarnitine	No: Undetected >80%		
	3-OH-oleylcarnitine	No: Undetected >80%		
Fatty acids	C14:0	Yes	--	B
	C15:0	Yes	Negative	A
	C16:0	Yes	Positive	B
	C16:1n7c	Yes	Positive	B
	C17:0	Yes	Negative	A
	C18:0	Yes	Negative	A
	C18:1n6c	Yes	Negative	A
	C18:1n6-9t	Yes	Negative	A
	C18:1n7c	Yes	Negative	A
	C18:1n9c	Yes	Positive	B
	C18:2n6t/t	Yes	Negative	A
	C18:2n6c/t	Yes	Negative	A
	C18:2n6t/c	Yes	--	A
	C18:2n6c/c	Yes	Positive	B
	C18:3n3	Yes	Positive	B
	C18:3n6	Yes	--	A
	C20:0	Yes	--	B
	C20:1n9	Yes	Negative	A
	C20:2n6	Yes	Negative	A
	C20:3n6	Yes	--	A
	C20:4n6 (Arachidonic acid (AA))	Yes	Negative	A
	C20:5n3 (Eicosapentaenoic acid (EPA))	Yes	--	A
	C22:0	Yes	Positive	B
	C22:4n6	Yes	--	A
	C22:5n3	Yes	Negative	A
	C22:5n6	Yes	Positive	B
	C22:6n3 (Docosahexaneonic acid (DHA))	Yes	Negative	A
	C24:0	Yes	Negative	A
	C24:1n9	Yes	Positive	B
	Σ(trans fatty acids)	Yes	Negative	A

	Σ(18:2 trans fatty acids)	Yes	--	A
	Σ(cis fatty acids)	Yes	Positive	B
	Σ(18:1 cis fatty acids)	Yes	Positive	B
	Σ(EPA, DHA)	Yes	Negative	A
	AA / EPA	Yes	--	A
	AA / DHA	Yes	Negative	A
	AA / Σ(EPA, DHA)	Yes	Negative	A
Amino Acids	1-Methylhistidine	No: 0s in >50%		
	3-Methylhistidine	No: 0s in >50%		
	α-aminobutyric acid	Yes	Negative	A
	Alanine	Yes	Positive	B
	Amino-Adipic Acid	No: 0s in >50%		
	Anserine	No: 0s in >50%		
	Arginine	Yes	Negative	A
	Asparagine	Yes	Positive	B
	Aspartic Acid	Yes	--	A
	β-alanine	No: 0s in >50%		
	β-aminoisobutyric acid	No: 0s in >50%		
	Carnosine	No: 0s in >50%		
	Citrulline	Yes	--	B
	Cystathionine	No: 0s in >50%		
	Cystine	Yes	--	B
	Ethanolamine	No: 0s in >50%		
	γ-aminobutyric acid	No: 0s in >50%		
	Glutamic Acid	Yes	--	A
	Glutamine	Yes	Positive	B
	Σ(Glutamic acid, Glutamine)	Yes	--	B
	Glycine	Yes	Negative	A
	Histidine	Yes	Positive	B
	Hydroxyproline	Yes	Positive	B
	Isoleucine	Yes	Negative	A
	Leucine	Yes	Negative	A

	Lysine	Yes	--	B
	Methionine	Yes	Positive	B
	OH-Lysine	No: 0s in >50%		
	Ornithine	Yes	--	B
	Phenylalanine	Yes	--	B
	Proline	Yes	Positive	B
	Sarcosine	No: 0s in >50%		
	Serine	Yes	--	B
	Taurine	Yes	Negative	A
	Threonine	Yes	Positive	B
	Tyrosine	Yes	--	B
	Valine	Yes	Negative	A

Fatty acids are formatted as C(number of carbons):(number of double bonds)n(location of first double bond). Where needed, fatty acid names are followed by c and/or t to denote cis or trans bonds.

Supplemental Table S2. Linear trajectories for metabolites with significant interactions. Betas show the estimated change in metabolite concentration per gestational week for each fetal sex, maternal race/ethnicity, and maternal BMI, using estimates from linear mixed effects models.

Metabolite	Stratified betas				Interaction p	FDR p
**Stratified by fetal sex**	**β (Females)**	**β (Males)**				
**C15:0 (%)**	-0.0012	-0.0021*			0.031	0.66
**C22:0 (%)**	0.0056	0.0004*			0.046	0.66
**Stearoylcarnitine (nmol/L)**	-0.0056	-0.23*			0.020	0.66
**Propionylcarnitine (nmol/L)**	-1.30	-2.41*			0.046	0.66
**Stratified by race/ethnicity**	**β (NHW)**	**β (NHB)**	**β (Hisp)**	**β (API)**		
**C20:3n6 (%)**	-0.0003	-0.0205**	-0.0057	0.0073	0.0042	0.16
**C20:2n6 (%)**	-0.0022	0.0001*	-0.0034	-0.0016	0.0054	0.23
**C18:1n7c (%)**	-0.0098	-0.0038**	-0.0089	-0.0097	0.0091	0.23
**C22:0 (%)**	0.0094	0.0013*	0.0008*	-0.0004*	0.021	0.40
**C18:3n6 (%)**	0.0001	-0.0012**	-0.0002	0.0000	0.027	0.41
**C20:4n6(%)**	-0.0873	-0.0525**	-0.0771	-0.0822	0.042	0.50
**Proline (umol/dL)**	-0.021	0.1139*	0.0885*	0.0969*	0.047	0.50
**Stratified by BMI**	**β (<25.0)**	**β (25.0-29.9)**	**β ≥30.0)**			
**C20:3n6 (%)**	-0.0005	-0.0014	-0.0297**		0.0016	0.11
**C17:0 (%)**	-0.0044	-0.0031*	-0.0018**		0.0028	0.11
**C16:1n7c (%)**	0.0065	0.0035	-0.0010**		0.022	0.42
**C16:0 (%)**	0.1100	0.0815*	0.0774		0.039	0.50
**AA/ Σ(EPA+DHA) (%)**	-0.0153	-0.0095	-0.0009*		0.046	0.50
**Dodecenoylcarnitine (nmol/L)**	-0.04	-0.54**	-0.45		0.017	0.42
**Alanine (umol/dL)**	0.2940	0.1798	0.0864*		0.030	0.46

** P < 0.05; ** P < 0.01; Abbreviations: NHW: Non-Hispanic White; NHB: Non-Hispanic Black; Hisp: Hispanic; API: Asian/Pacific Islander; FDR: Benjamini-Hochberg false discovery rate; BMI: body mass index. Betas stratified by fetal sex are compared to female fetuses; betas stratified by race/ethnicity are compared to NHW; betas stratified by BMI are compared to BMI <25.0*

Supplemental Table S3. Linear trajectories for metabolites with significant differences in slope or intercept. Intercepts are estimated levels of each metabolite at baseline (first visit), and slopes are estimated change in concentration per gestational week for each fetal sex, race/ethnicity, and BMI. Estimates from linear mixed effects models.

**Metabolite**	**Intercept**	**Slope**
Acylcarnitines		
**Propionylcarnitine (nmol/L)**		
Male	240.02	-2.41
Female	207.00	-1.30
Unadjusted p-value	*0.024*	*0.046*
**Octenoylcarnitine (nmol/L)**		
Normal BMI	75.18	0.04
Overweight BMI	103.10	-0.72
Obese BMI	104.49	-0.40
Unadjusted p-value	*0.0087*	*0.20*
**Dodecenoylcarnitine (nmol/L)**		
Normal BMI	20.86	-0.04
Overweight BMI	37.04	-0.54
Obese BMI	33.18	-0.45
**Stearoylcarnitine (nmol/L)**		
Male	27.37	-0.23
Female	22.62	-0.01
Unadjusted p-value	*0.036*	*0.02*
**Phospholipid fatty acids**		
**C14:0 (%)**		
Non-Hispanic White	0.35	0.0005
Non-Hispanic Black	0.26	-0.0006
Hispanic	0.31	0.0005
Asian/Pacific Islander	0.28	0.0017
Unadjusted p-value	*0.0052*	*0.27*
**C15:0 (%)**		
Male	0.29	-0.0021
Female	0.26	-0.0012
Unadjusted p-value	*0.011*	*0.031*
**C16:0 (%)**		
Normal BMI	25.81	0.1100
Overweight BMI	26.83	0.0815
Obese BMI	27.01	0.0774
Unadjusted p-value	*0.0039*	*0.039*
**C16:1n7c (%)**		
Normal BMI	0.43	0.0065
Overweight BMI	0.47	0.0035
Obese BMI	0.59	-0.0010
Unadjusted p-value	*0.038*	*0.022*

**C17:0 (%)**		
Normal BMI	0.51	-0.0044
Overweight BMI	0.48	-0.0031
Obese BMI	0.44	-0.0018
Unadjusted p-value	*0.0011*	*0.0028*
**C18:0 (%)**		
Non-Hispanic White	13.55	-0.0767
Non-Hispanic Black	13.84	-0.0579
Hispanic	13.56	-0.0586
Asian/Pacific Islander	12.70	-0.0510
Unadjusted p-value	*0.0023*	*0.081*
**18:1n6-9t (%)**		
Non-Hispanic White	0.91	-0.0068
Non-Hispanic Black	0.86	-0.0060
Hispanic	0.78	-0.0045
Asian/Pacific Islander	0.66	-0.0046
Unadjusted p-value	*0.0029*	*0.77*
**18:1n7c (%)**		
Non-Hispanic White	1.56	-0.0098
Non-Hispanic Black	1.40	-0.0038
Hispanic	1.57	-0.0089
Asian/Pacific Islander	1.64	-0.0097
Unadjusted p-value	*0.0005*	*0.0091*
**18:1n9c(%)**		
Non-Hispanic White	7.24	0.0370
Non-Hispanic Black	6.59	0.0291
Hispanic	6.73	0.0392
Asian/Pacific Islander	7.58	0.0303
Unadjusted p-value	*0.0015*	*0.79*
**18:3n6 (%)**		
Non-Hispanic White	0.08	0.0001
Non-Hispanic Black	0.10	-0.0012
Hispanic	0.08	-0.0002
Asian/Pacific Islander	0.07	0.0000
Unadjusted p-value	*0.08*	*0.027*
**20:2n6 (%)**		
Non-Hispanic White	0.53	-0.0022
Non-Hispanic Black	0.43	-0.0001
Hispanic	0.61	-0.0034
Asian/Pacific Islander	0.57	-0.0016
Unadjusted p-value	*<.0001*	*0.0054*
**20:3n6 (%)**		
Non-Hispanic White	3.54	-0.0003
Non-Hispanic Black	3.30	-0.0205
Hispanic	3.78	-0.0057

Asian/Pacific Islander	3.08	0.0073
Unadjusted p-value	*0.004*	*0.0042*
Normal BMI	3.32	-0.0005
Overweight BMI	3.44	-0.0014
Obese BMI	4.38	-0.0297
Unadjusted p-value	*<.0001*	*0.0016*
**20:4n6 (%)**		
Non-Hispanic White	12.19	-0.0873
Non-Hispanic Black	13.19	-0.0525
Hispanic	11.96	-0.0771
Asian/Pacific Islander	11.34	-0.0822
Unadjusted p-value	*0.0017*	*0.042*
**22:0 (%)**		
Non-Hispanic White	0.55	0.0094
Non-Hispanic Black	0.71	0.0013
Hispanic	0.60	0.0008
Asian/Pacific Islander	0.56	-0.0004
Unadjusted p-value	*0.34*	*0.021*
Male	0.61	0.0004
Female	0.58	0.0056
Unadjusted p-value	*0.64*	*0.046*
**22:5n3 (%)**		
Normal BMI	0.81	-0.0061
Overweight BMI	0.71	-0.0043
Obese BMI	0.71	-0.0071
Unadjusted p-value	*0.035*	*0.32*
**22:5n6 (%)**		
Non-Hispanic White	0.49	0.0025
Non-Hispanic Black	0.56	0.0016
Hispanic	0.52	0.0045
Asian/Pacific Islander	0.40	0.0022
Unadjusted p-value	*0.033*	*0.64*
**22:6n3 (%)**		
Non-Hispanic White	4.27	-0.0053
Non-Hispanic Black	4.25	-0.0107
Hispanic	4.04	-0.0140
Asian/Pacific Islander	5.30	-0.0132
Unadjusted p-value	*0.0003*	*0.82*
**Σ(trans fatty acids) (%)**		
Non-Hispanic White	1.12	-0.0070
Non-Hispanic Black	1.10	-0.0071
Hispanic	1.00	-0.0052
Asian/Pacific Islander	0.86	-0.0057
Unadjusted p-value	*0.006*	*0.89*
**Σ(cis fatty acids) (%)**		

Non-Hispanic White	29.56	0.0635
Non-Hispanic Black	27.70	0.1114
Hispanic	29.02	0.0964
Asian/Pacific Islander	30.48	0.0814
Unadjusted p-value	*0.0022*	*0.15*
Normal BMI	29.61	0.0853
Overweight BMI	29.44	0.0817
Obese BMI	27.70	0.1122
Unadjusted p-value	*0.023*	*0.51*
**Σ(18:1 cis fatty acids) (%)**		
Non-Hispanic White	9.02	0.0276
Non-Hispanic Black	8.22	0.0239
Hispanic	8.51	0.0296
Asian/Pacific Islander	9.40	0.0198
Unadjusted p-value	*0.0007*	*0.88*
**Σ(EPA, DHA) (%)**		
Non-Hispanic White	4.63	-0.0060
Non-Hispanic Black	4.55	-0.0117
Hispanic	4.33	-0.0157
Asian/Pacific Islander	5.55	-0.0139
Unadjusted p-value	*0.0008*	*0.78*
**AA / DHA (%)**		
Non-Hispanic White	3.08	-0.0203
Non-Hispanic Black	3.30	-0.0079
Hispanic	3.16	-0.0117
Asian/Pacific Islander	2.26	-0.0104
Unadjusted p-value	*<.0001*	*0.28*
**AA / Σ(EPA, DHA) (%)**		
Non-Hispanic White	2.81	-0.0180
Non-Hispanic Black	3.05	-0.0064
Hispanic	2.94	-0.0102
Asian/Pacific Islander	2.14	-0.0097
Unadjusted p-value	*<.0001*	*0.22*
Normal BMI	2.66	-0.0153
Overweight BMI	2.79	-0.0095
Obese BMI	2.86	-0.0009
Unadjusted p-value	*0.47*	*0.046*
**Amino acids**		
**Alanine (umol/dL)**		
Normal BMI	23.98	0.29
Overweight BMI	26.49	0.18
Obese BMI	26.18	0.09
Unadjusted p-value	*0.17*	*0.030*
**Σ(Glutamic acid, Glutamine) (umol/dL)**		
Male	41.94	0.10

Female	44.80	0.00
Unadjusted p-value	*0.024*	*0.06*
**Glycine (umol/dL)**		
Non-Hispanic White	16.80	-0.06
Non-Hispanic Black	14.51	-0.00
Hispanic	14.42	-0.02
Asian/Pacific Islander	15.17	-0.05
Unadjusted p-value	*0.009*	*0.08*
Normal BMI	16.11	-0.05
Overweight BMI	14.79	-0.03
Obese BMI	12.69	0.00
Unadjusted p-value	*0.0002*	*0.26*
**Proline (umol/dL)**		
Non-Hispanic White	14.66	-0.02
Non-Hispanic Black	11.10	0.11
Hispanic	11.60	0.09
Asian/Pacific Islander	11.01	0.10
Unadjusted p-value	*0.023*	*0.047*
**Threonine (umol/dL)**		
Male	8.57	0.30
Female	10.13	0.23
Unadjusted p-value	*0.040*	*0.07*
Non-Hispanic White	8.16	0.28
Non-Hispanic Black	11.58	0.21
Hispanic	9.52	0.26
Asian/Pacific Islander	8.74	0.30
Unadjusted p-value	*0.017*	*0.40*

Abbreviations: AA: arachidonic acid; BMI (body mass index); DHA: docosahexaneonic acid dL: deciliter; EPA: eicosapentaenoic acid; L: liter; nmol: nanomol; umol: micromol

Supplemental Table S4. Linear models with all data and after excluding the fasting visit.

	Linear models: all visits		Linear models: dropping fasting visit	
	Intercept	Slope	Intercept	Slope
Acylcarnitines (nmol/L)				
Acetylcarnitine	4040.6	-44.5**	4045.2	-43.8**
Propionylcarnitine	224.8	-1.9**	239.3	-2.2**
Octenoylcarnitine	88.5	-0.3	86.3	-0.3
Decenoylcarnitine	75.3	-0.7**	67.1	-0.6**
Decanoylcarnitine	94.1	-1.0 **	81.0	-0.7**
Glutarylcarnitine	19.6	-0.1**	18.0	-0.1*
Dodecenoylcarnitine	27.8	-0.3**	24.1	-0.2**
Dodecanoylcarnitine	39.6	-0.4**	36.5	-0.3**
Tetradecenoylcarnitine	30.0	-0.4**	27.3	-0.4**
Tetradecanoylcarnitine	16.5	-0.1	15.4	-0.0
Hexadecenoylcarnitine	13.1	-0.1**	12.5	-0.1**
Hexadecanoylcarnitine	57.7	-0.3 *	58.2	-0.3
Oleylcarnitine	64.3	-0.5**	65.9	-0.5*
Linoleylcarnitine	30.3	-0.3*	30.7	-0.3 *
Stearoylcarnitine	25.0	-0.1*	25.2	-0.1*
Phospholipid fatty acids (relative percent)				
C14:0	0.31	0.0005	0.30	0.0007
C15:0	0.28	-0.0016**	0.27	-0.0016**
C16:0	26.31	0.0960**	25.90	0.1035**
C16:1n7c	0.47	0.0045	0.46	0.0049**
C17:0	0.49	-0.0036**	0.49	-0.0038**
C18:0	13.42	-0.0622**	13.49	-0.0628**
C18:1n6c	0.22	-0.0013**	0.22	-0.0013**
C18:1n6/9t	0.80	-0.0055**	0.81	-0.0057**
C18:1n7c	1.55	-0.0080**	1.50	-0.0073**
C18:1n9c	7.03	0.0349**	6.88	0.0370**
C18:2n6c/c	19.95	0.0603**	19.60	0.0691**
C18:2n6c/t	0.08	-0.0004*	0.09	-0.0005**
C18:2n6t/c	0.08	-0.0000	0.08	-0.0000
C18:2n6t/t	0.06	-0.0003**	0.05	-0.0002*
C18:3n3	0.20	0.0020**	0.19	0.0023**
C18:3n6	0.08	-0.0003	0.08	-0.0002
C20:0	0.25	0.0006	0.31	-0.0007*
C20:1n9	0.15	-0.0009**	0.16	-0.0011**
C20:2n6	0.55	-0.0019**	0.54	-0.0021**
C20:3n6	3.50	-0.0043	3.48	-0.0040
C20:4n6	12.01	-0.0752**	12.19	-0.0780**
C20:5n3	0.30	-0.0010	0.37	-0.0025**
C22:0	0.59	0.0031*	0.78	-0.0009
C22:4n6	0.42	0.0007	0.54	-0.0023**
C22:5n3	0.76	-0.0056**	0.78	-0.0060**

C22:5n6	0.50	0.0028**	0.52	0.0024**
C22:6n3	4.41	-0.0104**	4.50	-0.0139**
C24:0	0.59	-0.0025**	0.60	-0.0025**
C24:1n9	0.78	0.0046*	1.09	-0.0021
Σ(trans fatty acids)	1.02	-0.0063**	1.03	-0.0065**
Σ(18:2 trans fatty acids)	0.21	-0.0006	0.22	-0.0006**
Σ(cis fatty acids)	29.28	0.0876**	28.71	0.0999**
Σ(18:1 cis fatty acids)	8.80	0.0261**	8.60	0.0283**
AA / DHA	2.96	-0.0136**	2.94	-0.0120**
AA/EPA	52.94	-0.1811	41.19	0.0670
AA/ Σ(EPA, DHA)	2.74	-0.0120**	2.70	-0.0102**
Σ(EPA, DHA)	4.71	-0.0114**	4.86	-0.0160**
Amino acids (umol/dL)				
α-aminobutyric acid	1.44	-0.0042*	1.48	-0.0046**
Alanine	25.04	0.2303**	25.68	0.2275**
Arginine	6.40	-0.0385**	5.68	-0.0213*
Asparagine	4.43	0.0339**	4.96	0.0249**
Aspartic acid	0.80	0.0000	0.65	0.0030*
Citrulline	1.71	0.0002	1.74	-0.0001
Cystine	1.01	0.0082	1.27	0.0018
Glutamic acid	13.83	-0.0472	11.56	0.0039
Glutamine	29.59	0.1088**	32.24	0.0449
Σ(Glutamic acid, Glutamine)	43.38	0.0516	43.91	0.0462
Glycine	15.19	-0.0352**	15.03	-0.0298**
Histidine	6.44	0.0363**	6.52	0.0372**
Hydroxyproline	0.99	0.0107**	1.05	0.0093*
Isoleucine	5.44	-0.0199*	5.61	-0.0224*
Leucine	9.77	-0.0477**	10.18	-0.0539**
Lysine	14.81	0.0104	14.92	0.0107
Methionine	1.73	0.0072**	1.73	0.0083**
Ornithine	2.77	-0.0042	3.06	-0.0079
Phenylalanine	4.98	0.0001	5.20	-0.0016
Proline	12.28	0.0629**	13.35	0.0431
Serine	7.87	0.0012	7.61	0.0071
Taurine	10.16	-0.0975**	10.39	-0.0936**
Threonine	9.35	0.2673**	9.14	0.2622**
Tyrosine	4.26	0.0061	4.53	0.0037
Valine	18.81	-0.0833**	19.47	-0.0878**

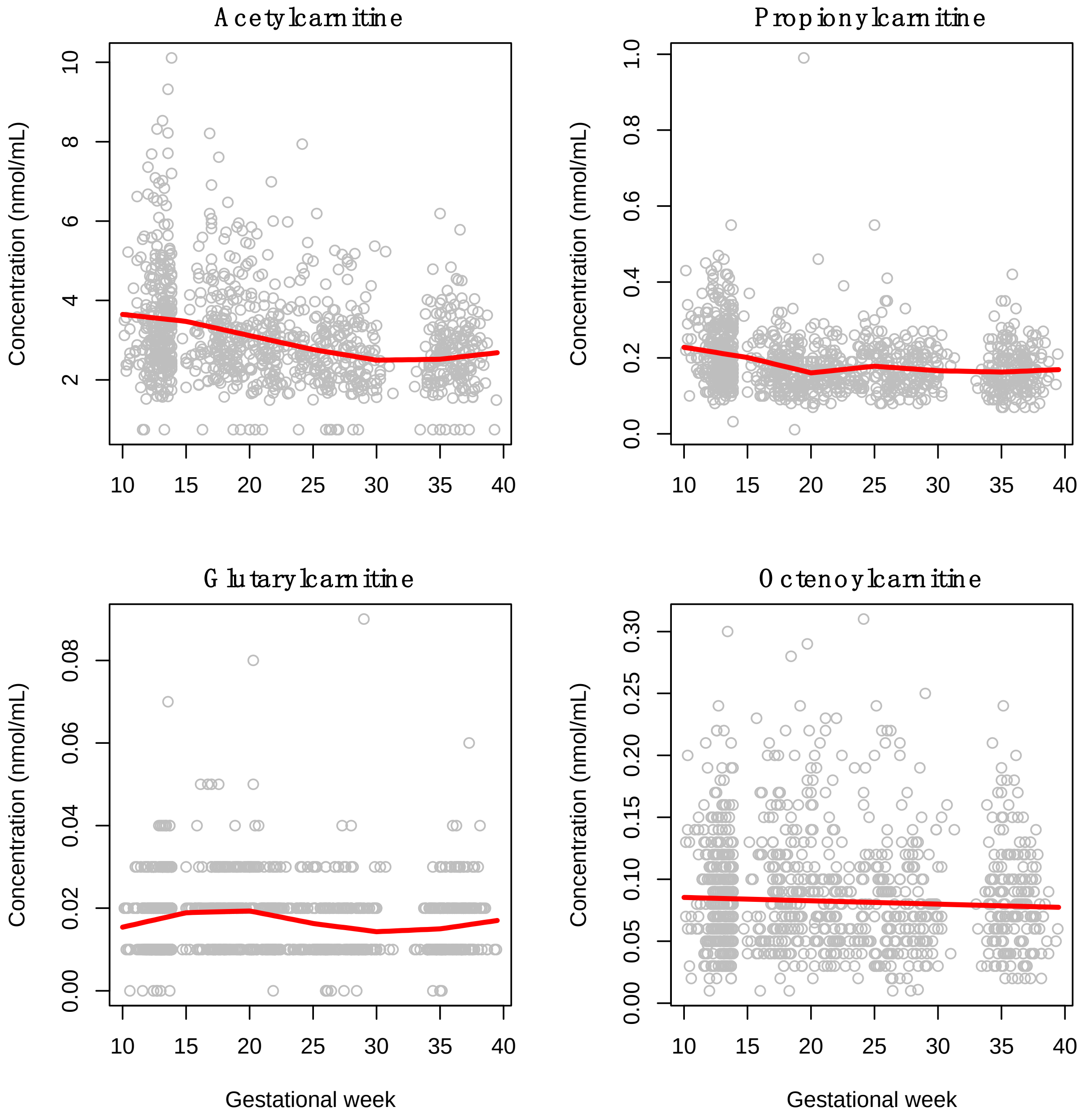

Acetylcarnitine
Propionylcarnitine
Glutarylcarnitine
Octenoylcarnitine
Concentration (nmol/mL)
Gestational week
10 15 20 25 30 35 40
2 4 6 8 10
0.0 0.2 0.4 0.6 0.8 1.0
0.00 0.02 0.04 0.06 0.08
0.00 0.05 0.10 0.15 0.20 0.25 0.30

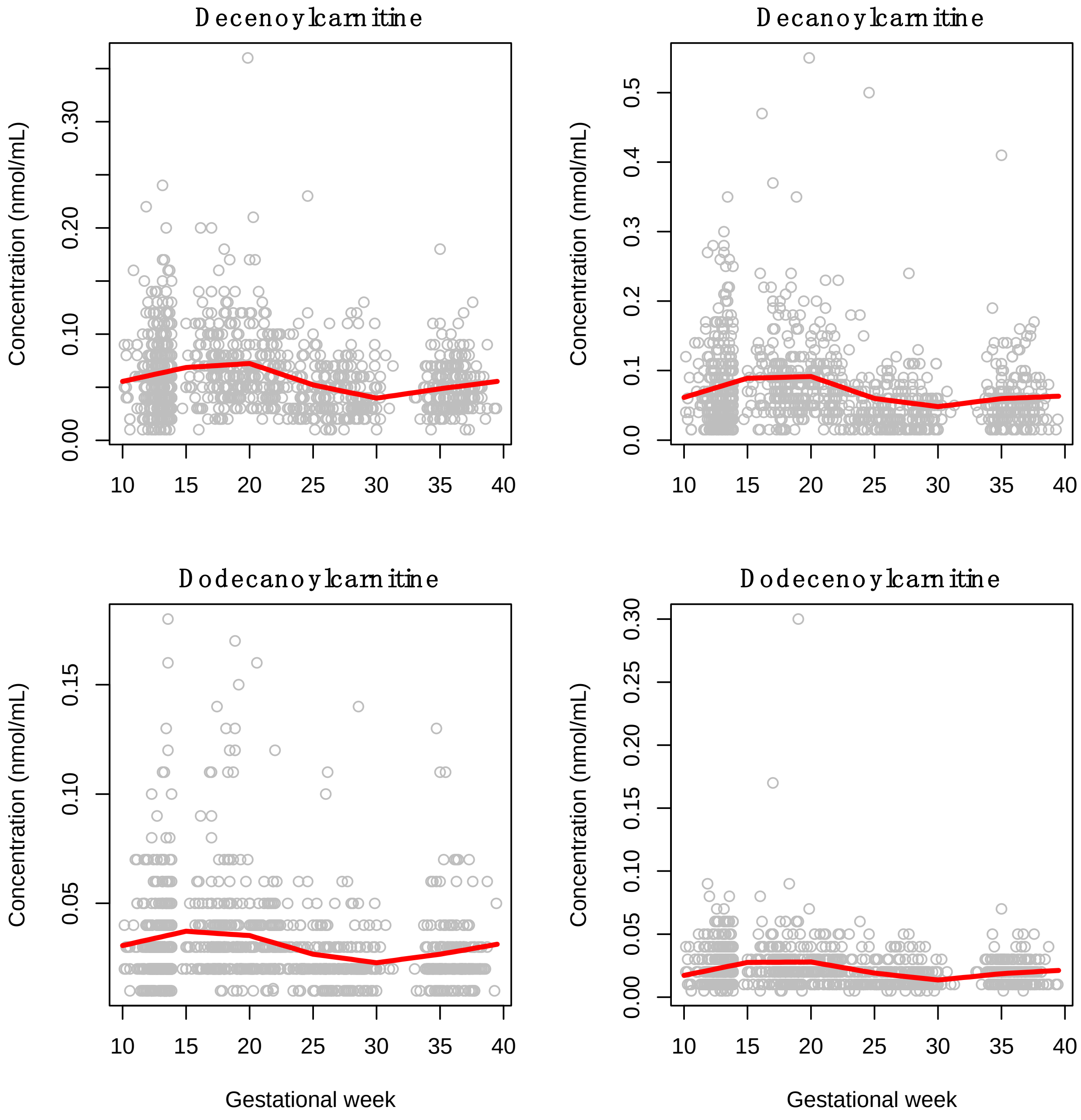

Decenoylcarnitine
Decanoylcarnitine
Dodecanoylcarnitine
Dodecenoylcarnitine
Concentration (nmol/mL)
Gestational week

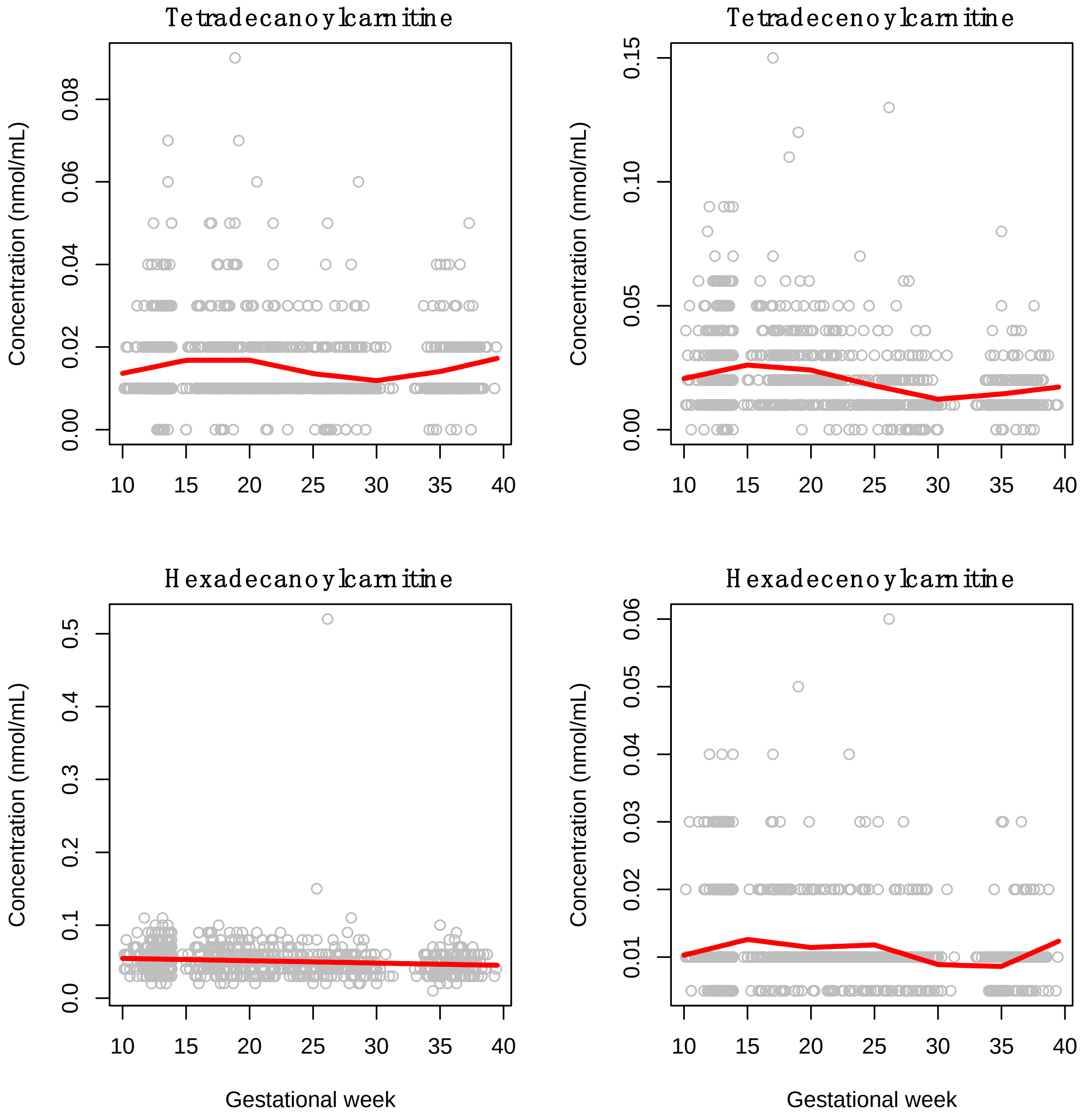
Tetradecanoylcarnitine
Tetradecenoylcarnitine
Hexadecanoylcarnitine
Hexadecenoylcarnitine
Concentration (nmol/mL)
Gestational week
10
15
20
25
30
35
40
0.00
0.02
0.04
0.06
0.08
0.05
0.10
0.15
0.0
0.1
0.2
0.3
0.4
0.5
0.01
0.03
0.06

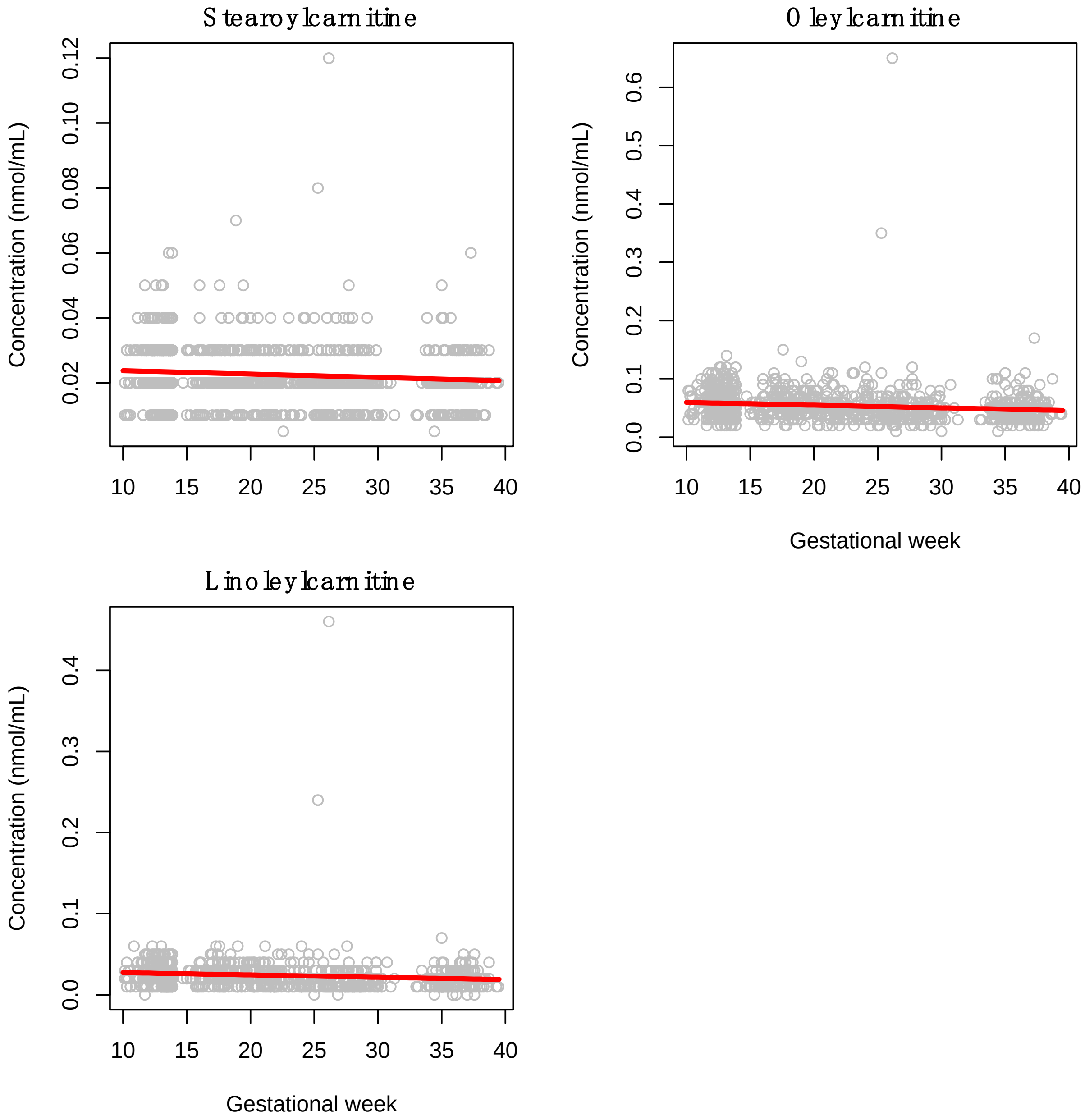
Stearoylcarnitine
Oleylcarnitine
Linoleylcarnitine
Concentration (nmol/mL)
Gestational week
10 15 20 25 30 35 40
0.02 0.04 0.06 0.08 0.10 0.12
0.0 0.1 0.2 0.3 0.4 0.5 0.6
0.0 0.1 0.2 0.3 0.4

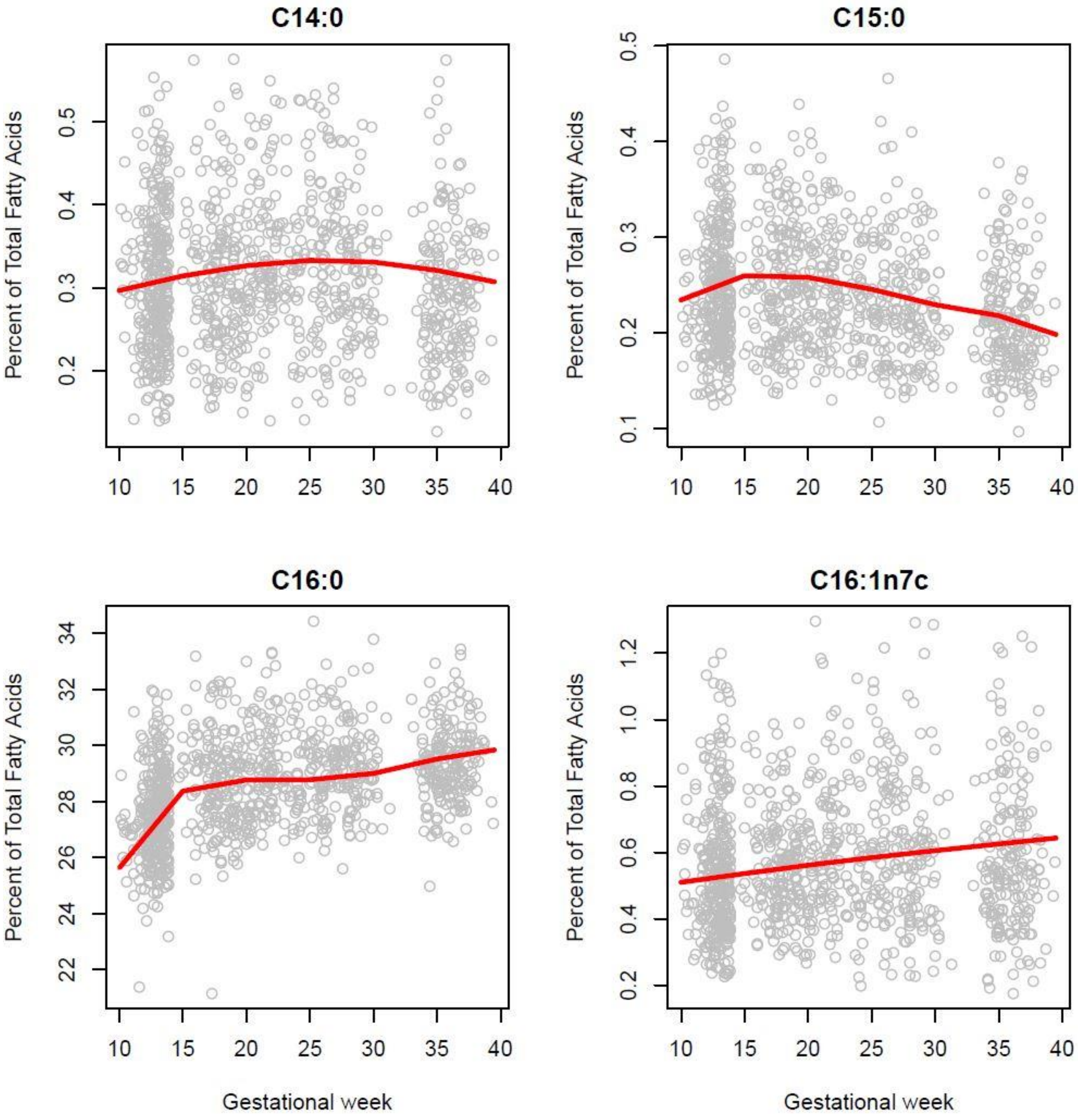
C14:0
C15:0
C16:0
C16:1n7c
Percent of Total Fatty Acids
Gestational week

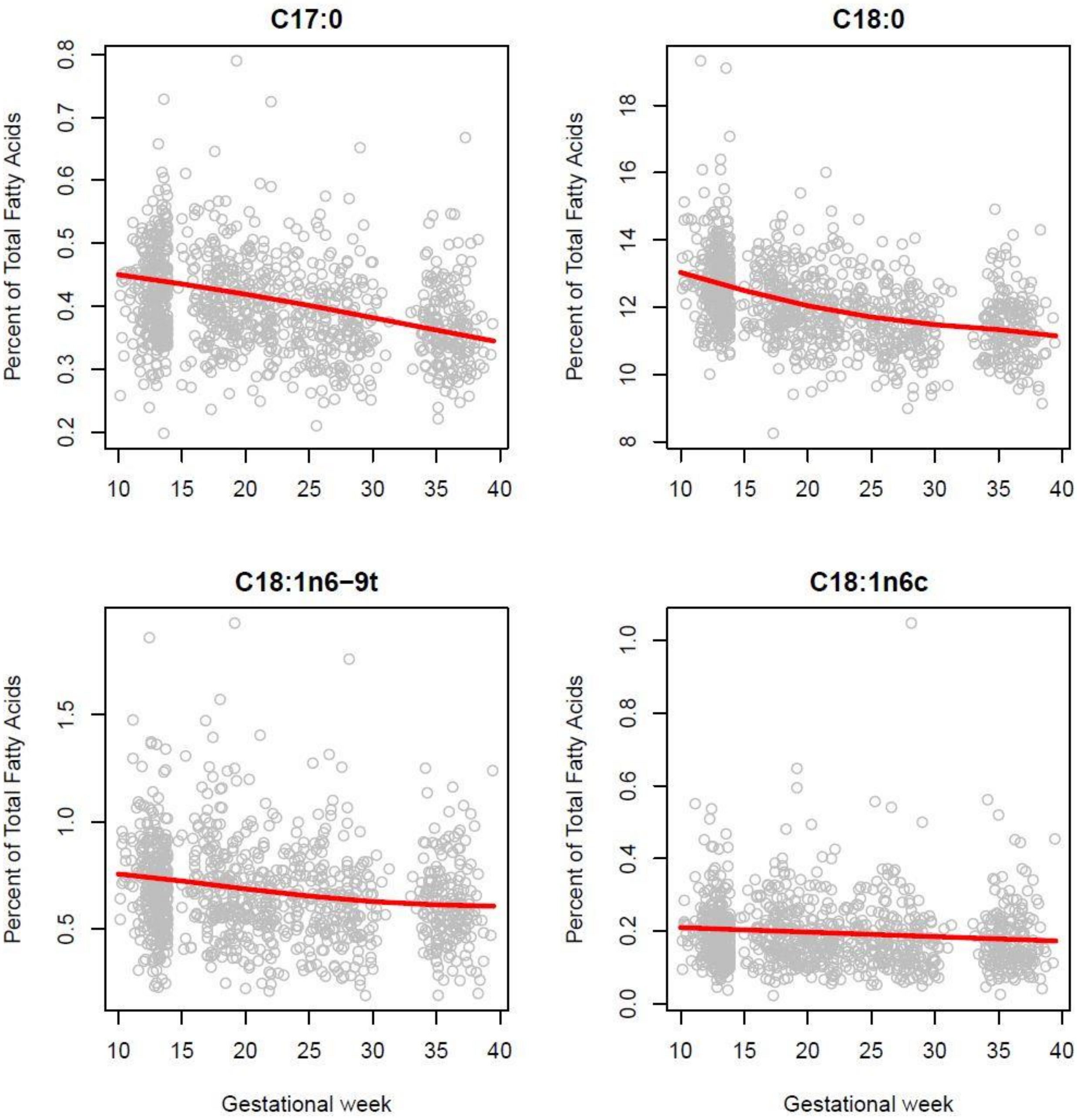
C17:0
Percent of Total Fatty Acids
0.2
0.3
0.4
0.5
0.6
0.7
0.8
10 15 20 25 30 35 40
C18:0
Percent of Total Fatty Acids
8
10
12
14
16
18
10 15 20 25 30 35 40
C18:1n6−9t
Percent of Total Fatty Acids
0.5
1.0
1.5
10 15 20 25 30 35 40
Gestational week
C18:1n6c
Percent of Total Fatty Acids
0.0
0.2
0.4
0.6
0.8
1.0
10 15 20 25 30 35 40
Gestational week

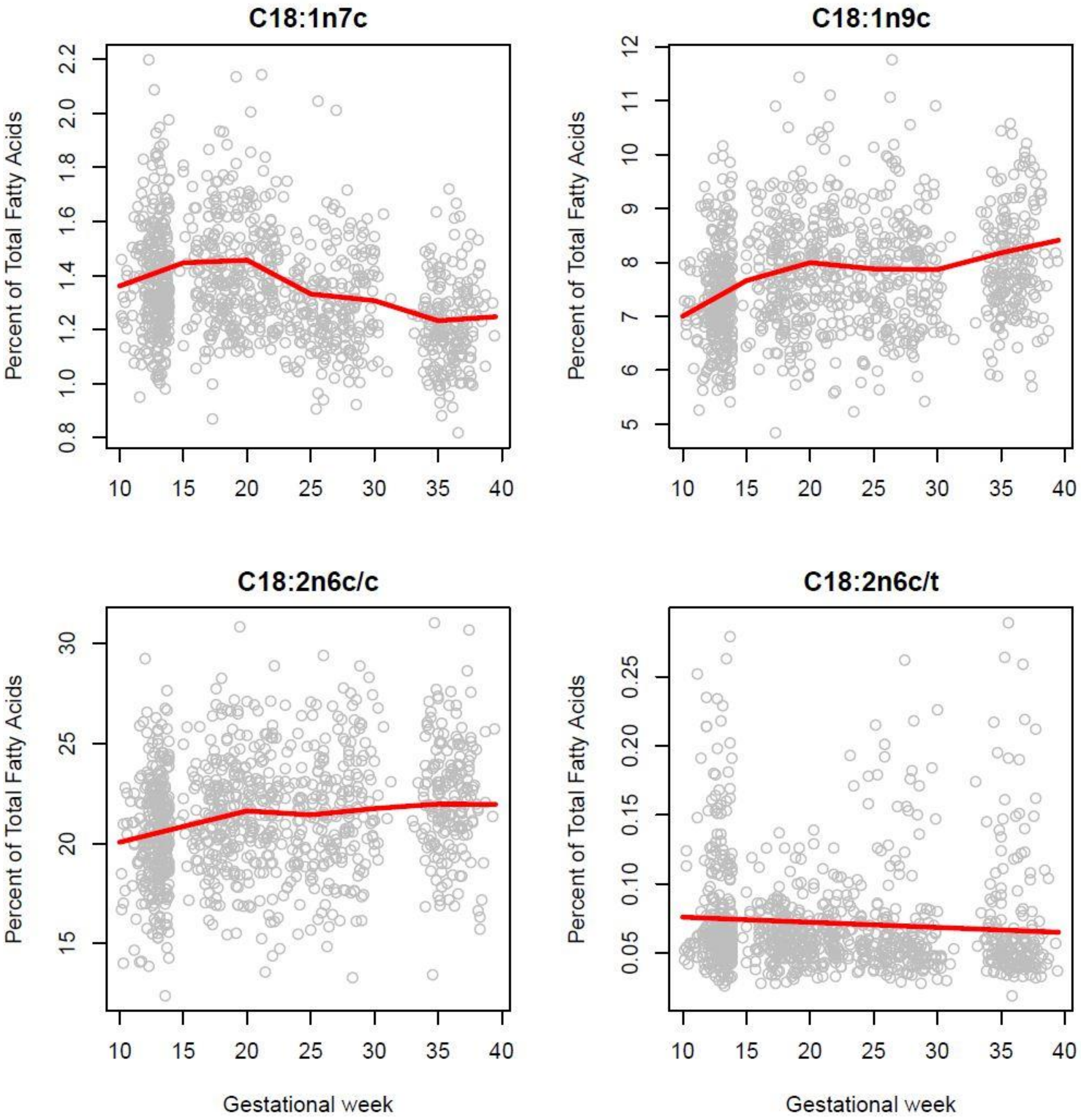
C18:1n7c
C18:1n9c
C18:2n6c/c
C18:2n6c/t
Percent of Total Fatty Acids
Gestational week

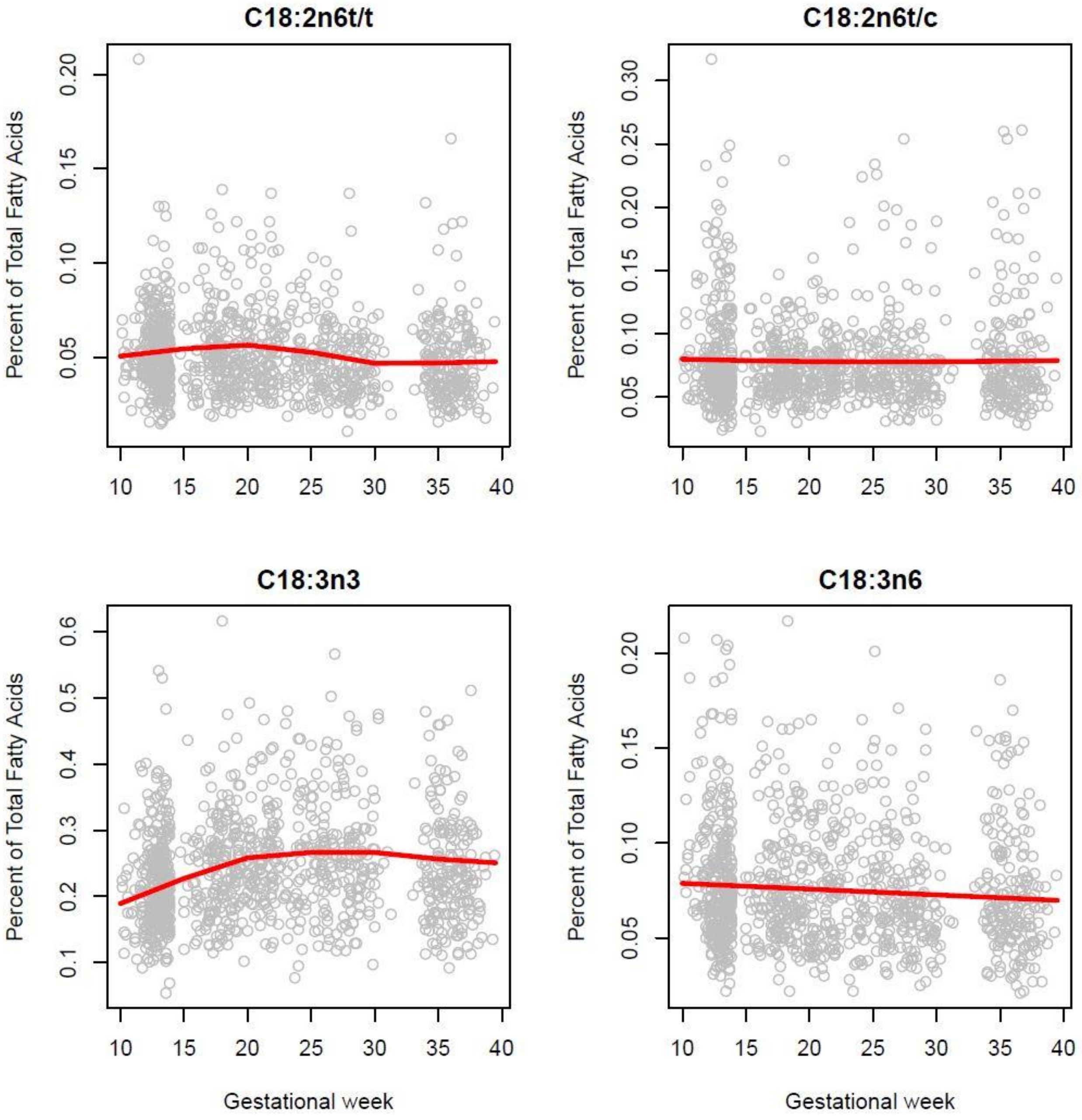
C18:2n6t/t
C18:2n6t/c
C18:3n3
C18:3n6
Percent of Total Fatty Acids
Gestational week
10
15
20
25
30
35
40
0.05
0.10
0.15
0.20
0.25
0.30
0.1
0.2
0.3
0.4
0.5
0.6

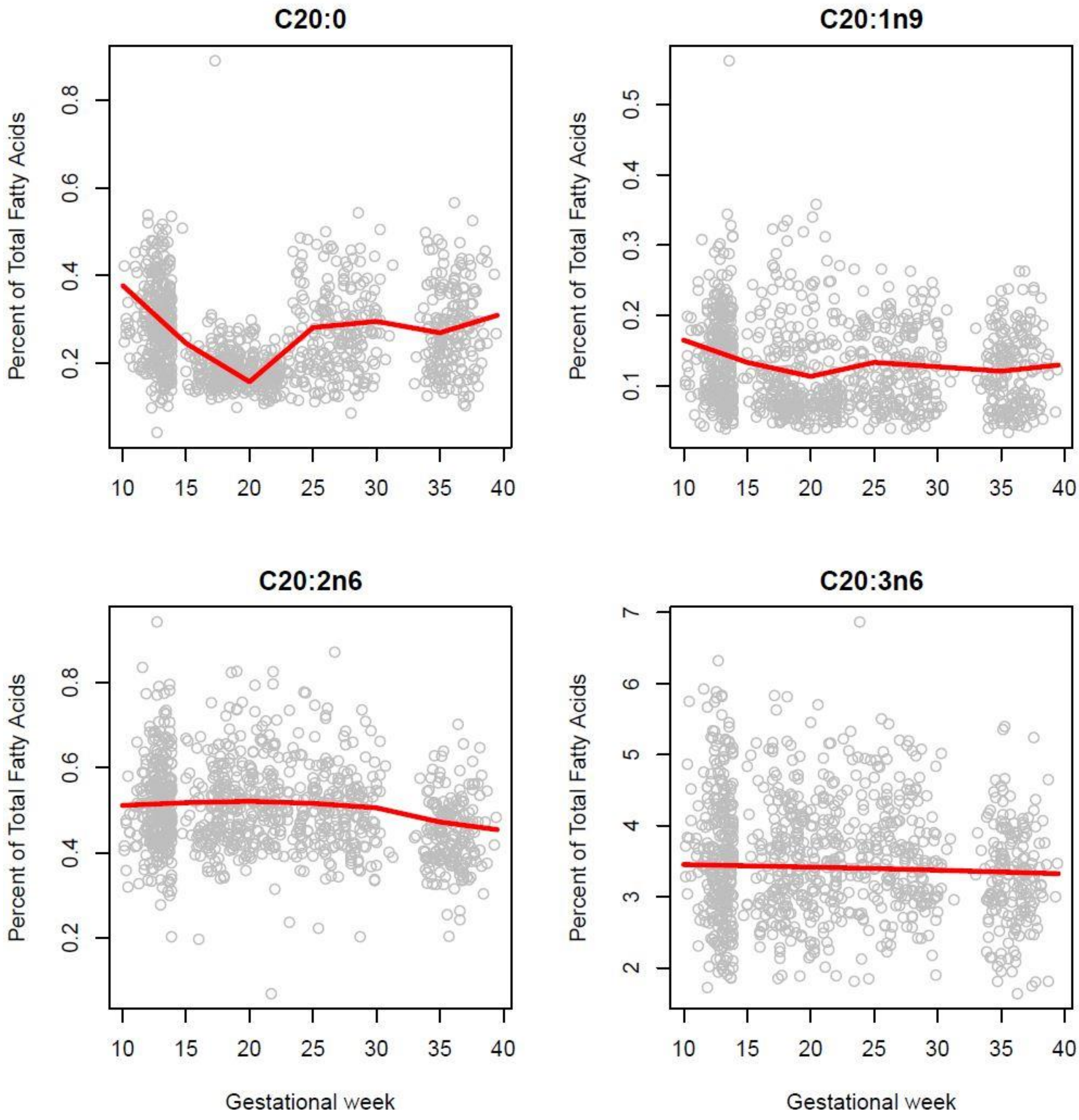
C20:0
C20:1n9
C20:2n6
C20:3n6
Percent of Total Fatty Acids
Gestational week
0.2
0.4
0.6
0.8
0.1
0.2
0.3
0.4
0.5
2
3
4
5
6
7
10
15
20
25
30
35
40

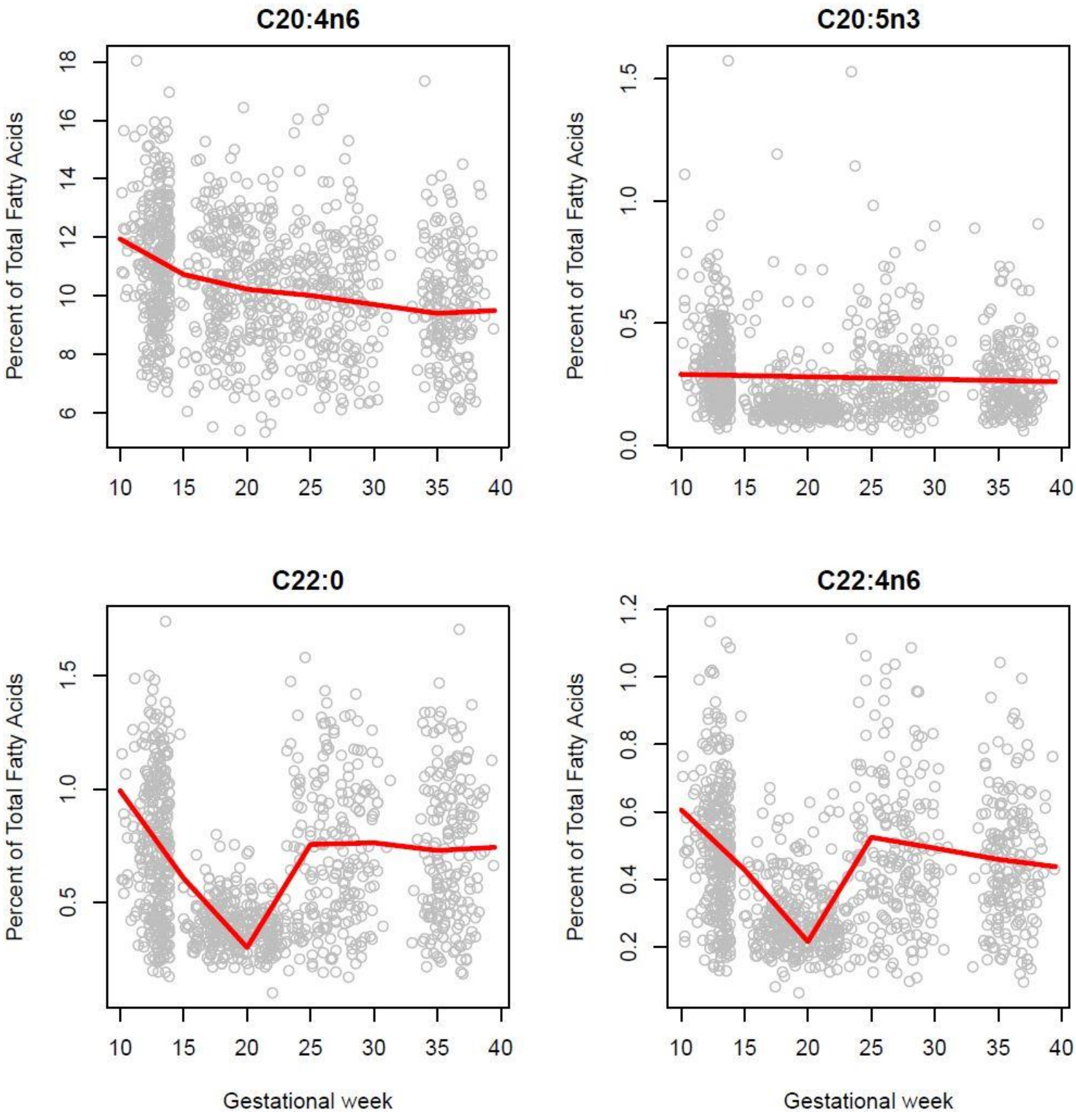
C20:4n6
C20:5n3
C22:0
C22:4n6
Percent of Total Fatty Acids
Gestational week
10
15
20
25
30
35
40

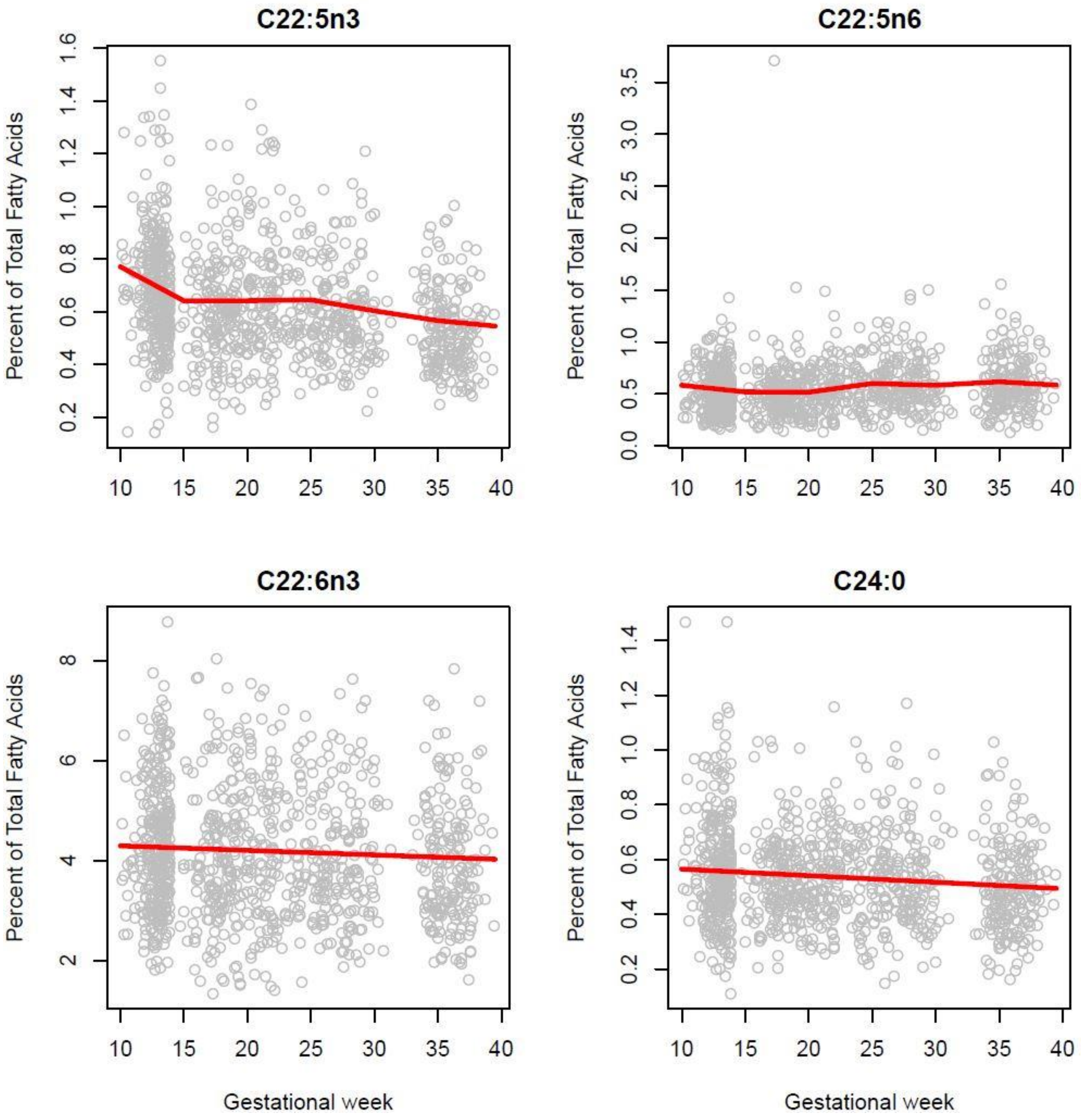
C22:5n3
C22:5n6
C22:6n3
C24:0
Percent of Total Fatty Acids
Gestational week
10 15 20 25 30 35 40
0.2 0.4 0.6 0.8 1.0 1.2 1.4 1.6
0.0 0.5 1.0 1.5 2.0 2.5 3.0 3.5
2 4 6 8
0.2 0.4 0.6 0.8 1.0 1.2 1.4

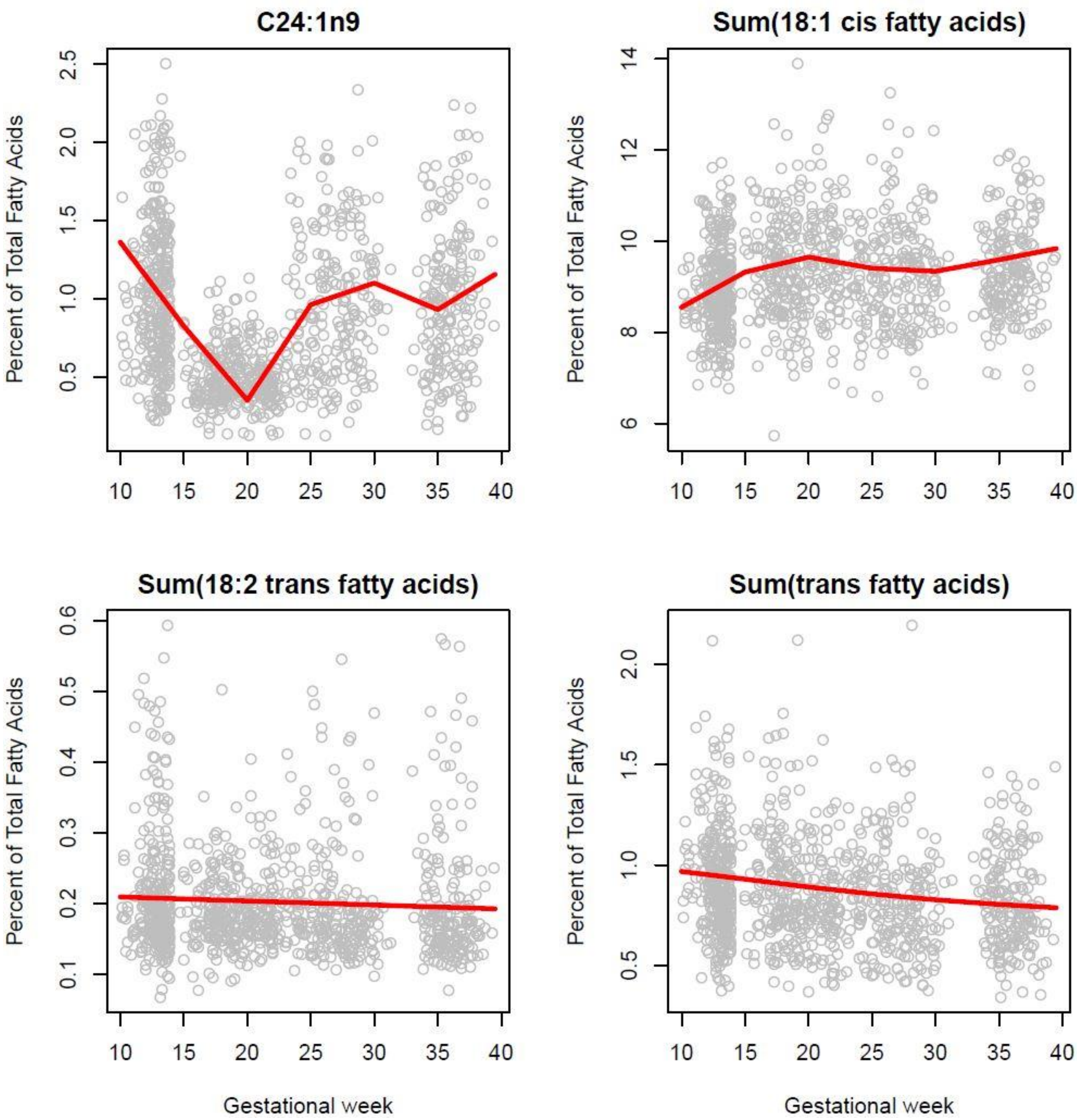
C24:1n9
Sum(18:1 cis fatty acids)
Sum(18:2 trans fatty acids)
Sum(trans fatty acids)
Percent of Total Fatty Acids
Gestational week

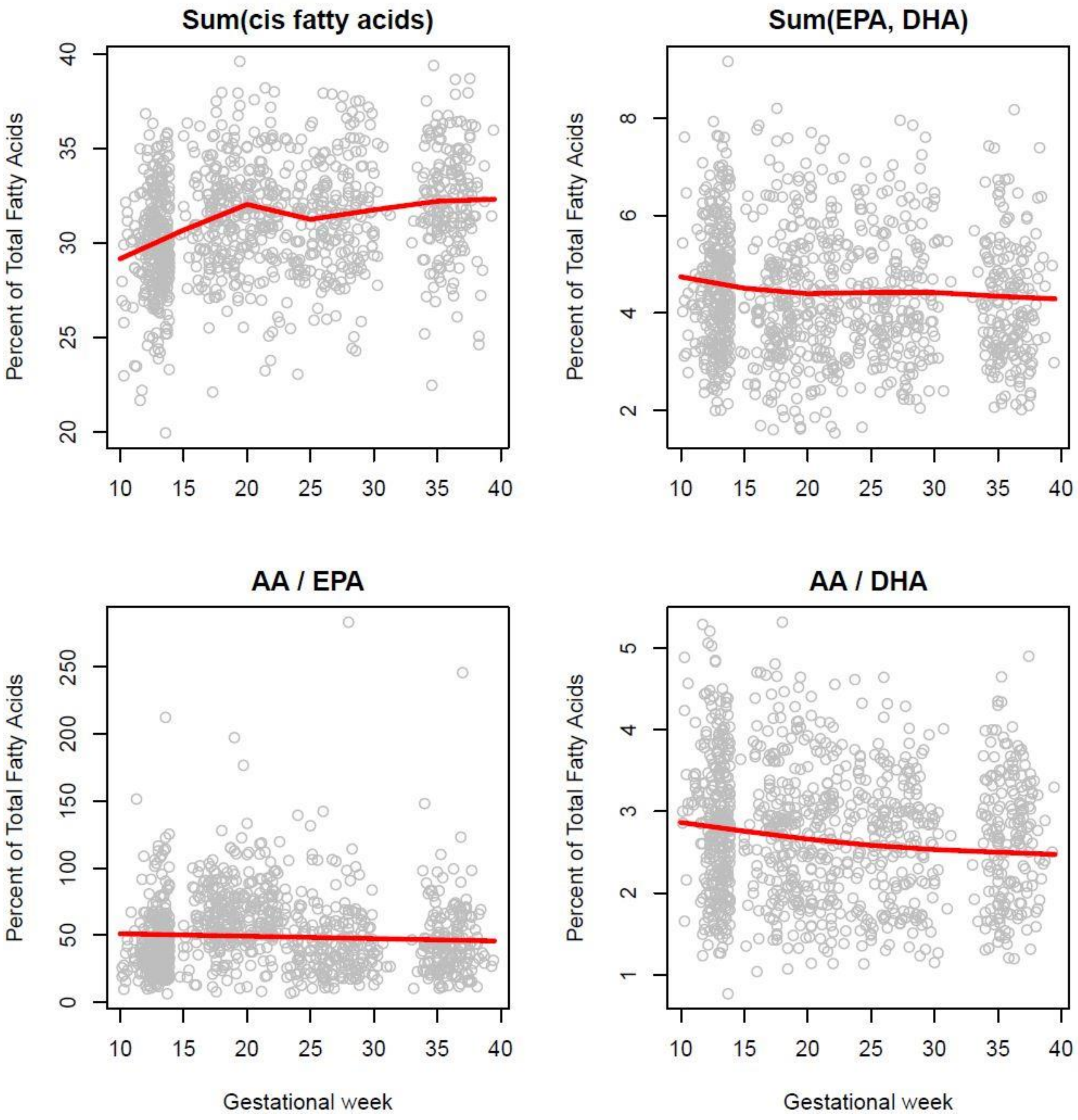
Sum(cis fatty acids)
Sum(EPA, DHA)
AA / EPA
AA / DHA
Percent of Total Fatty Acids
Gestational week

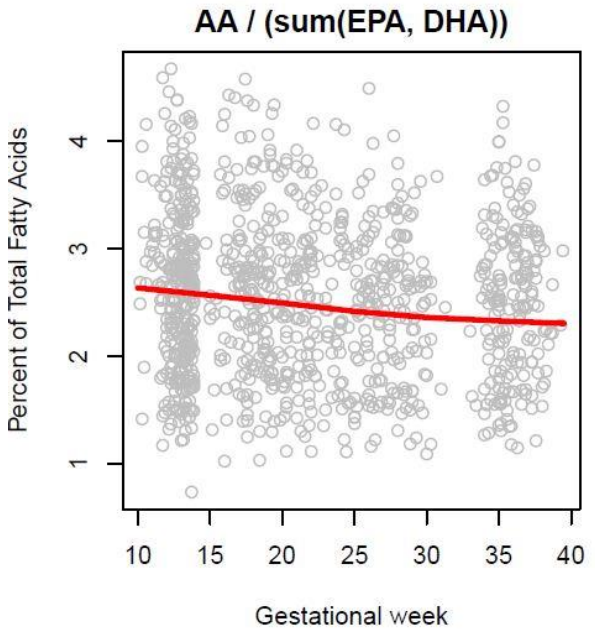
AA / (sum(EPA, DHA))
Percent of Total Fatty Acids
1
2
3
4
10
15
20
25
30
35
40
Gestational week

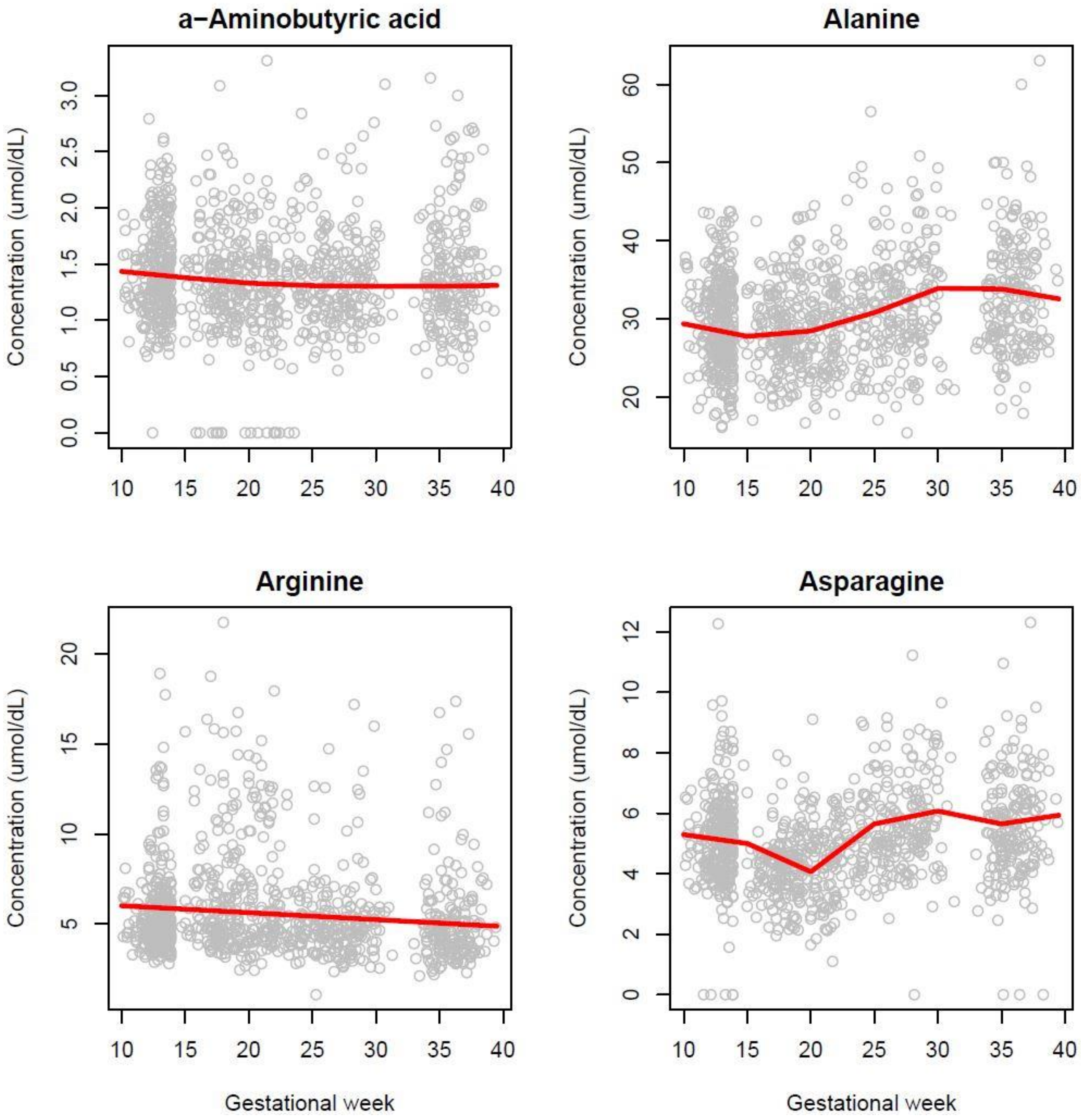
a−Aminobutyric acid
Alanine
Arginine
Asparagine
Concentration (umol/dL)
Gestational week

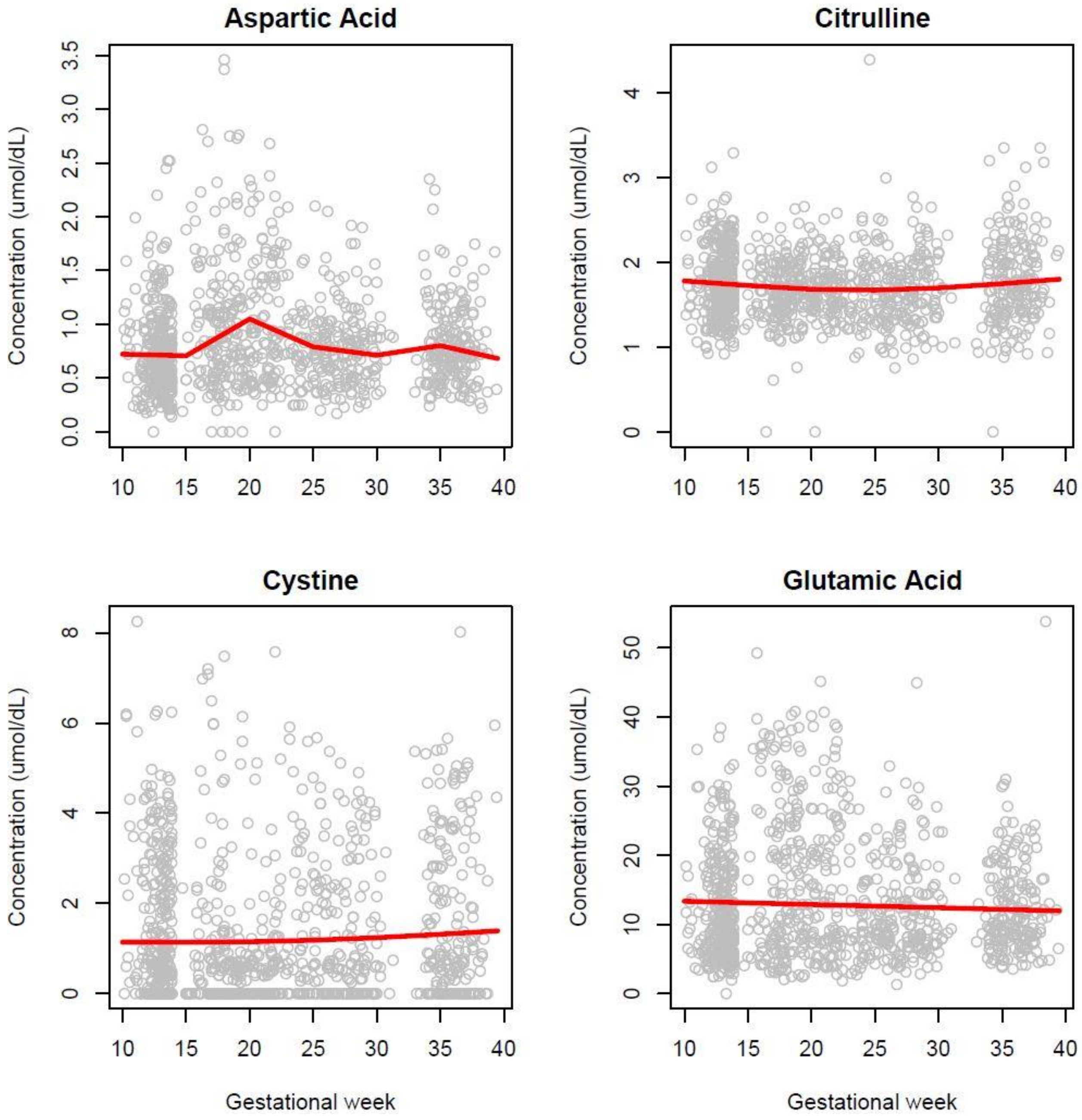

Aspartic Acid
Citrulline
Cystine
Glutamic Acid
Concentration (umol/dL)
Gestational week

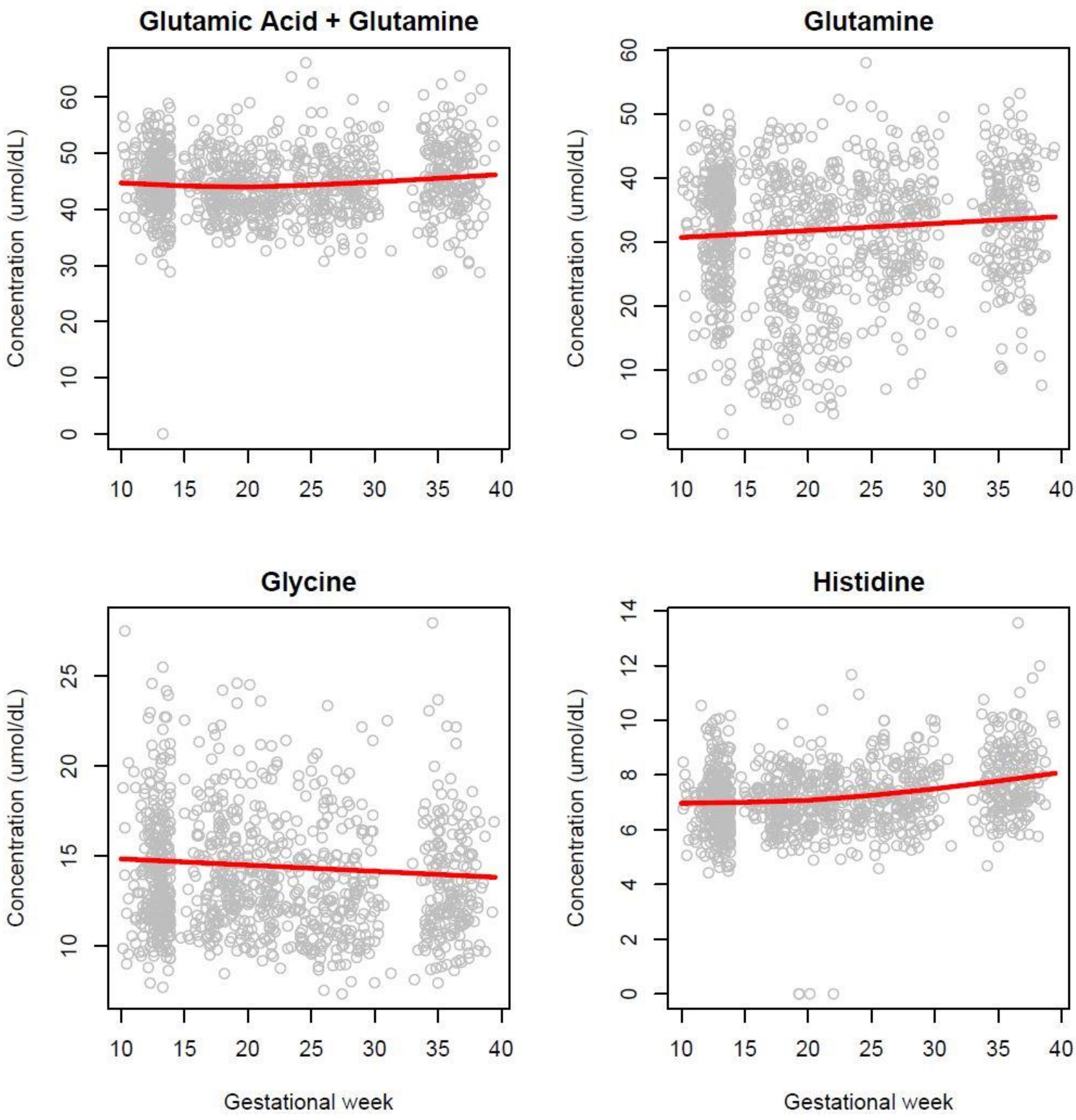
Glutamic Acid + Glutamine
Glutamine
Glycine
Histidine
Concentration (umol/dL)
Gestational week

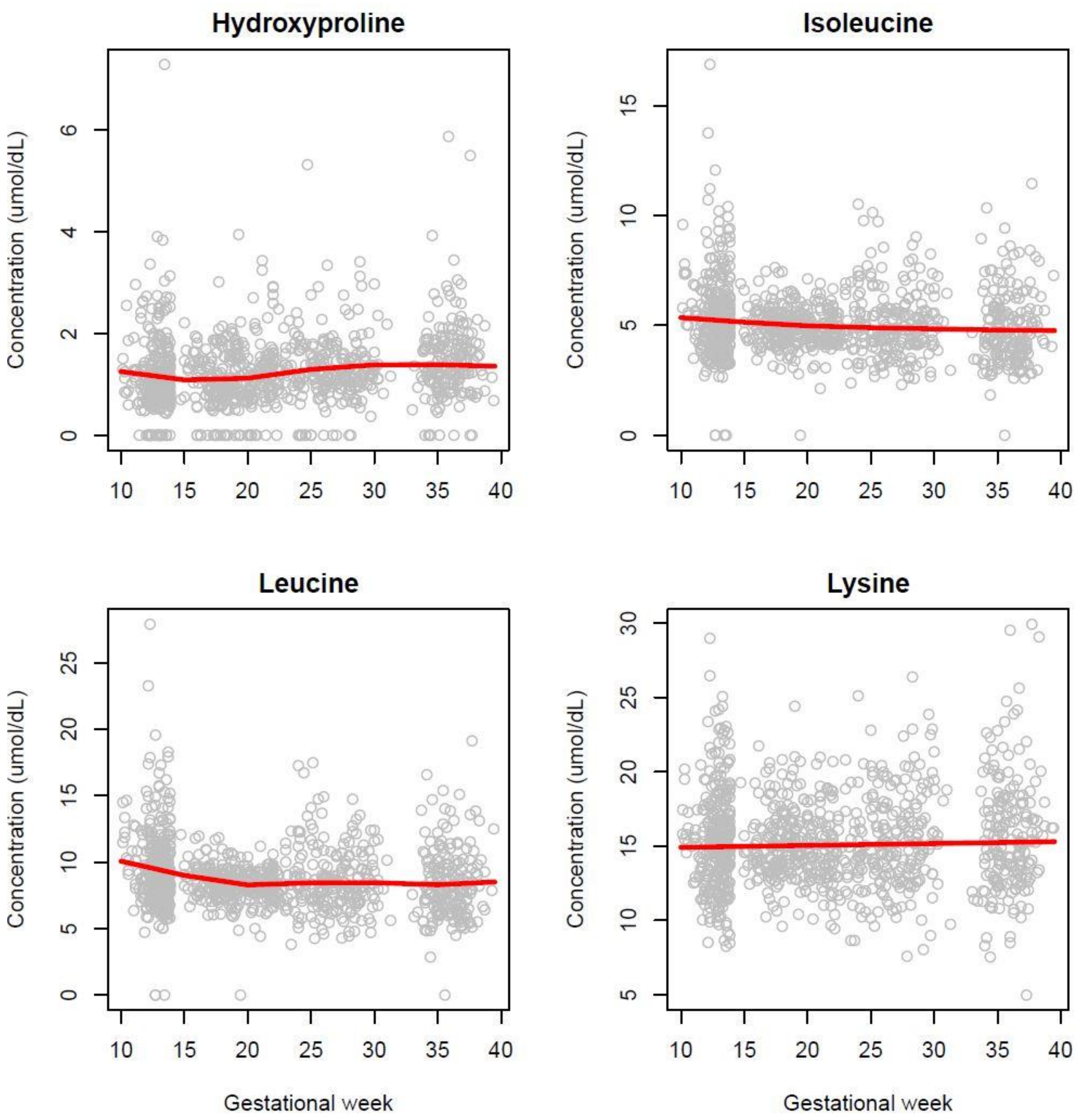
Hydroxyproline
Isoleucine
Leucine
Lysine
Concentration (umol/dL)
Gestational week
0
2
4
6
5
10
15
20
25
30
10
15
20
25
30
35
40

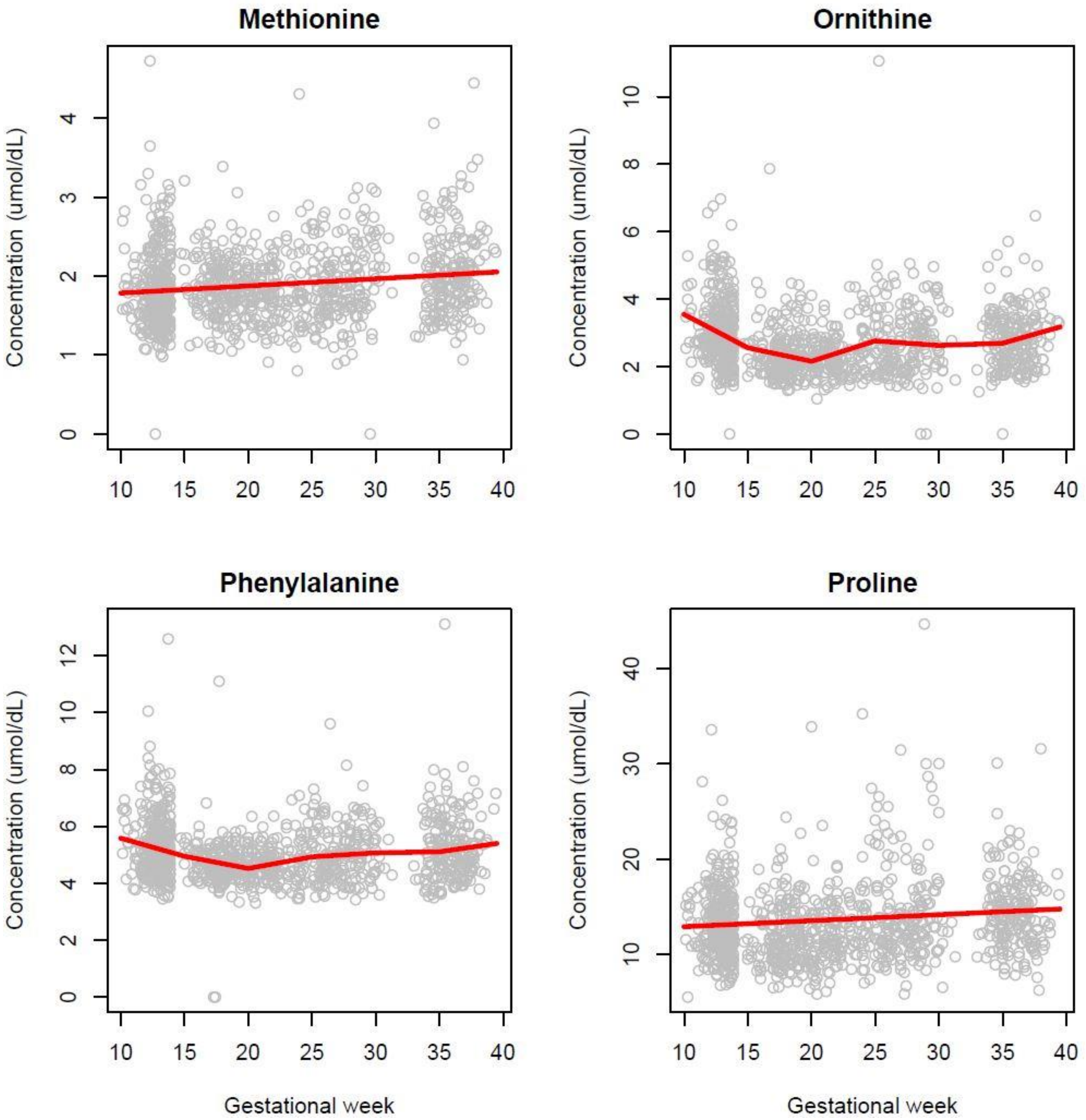

Methionine
Ornithine
Phenylalanine
Proline
Concentration (umol/dL)
Gestational week

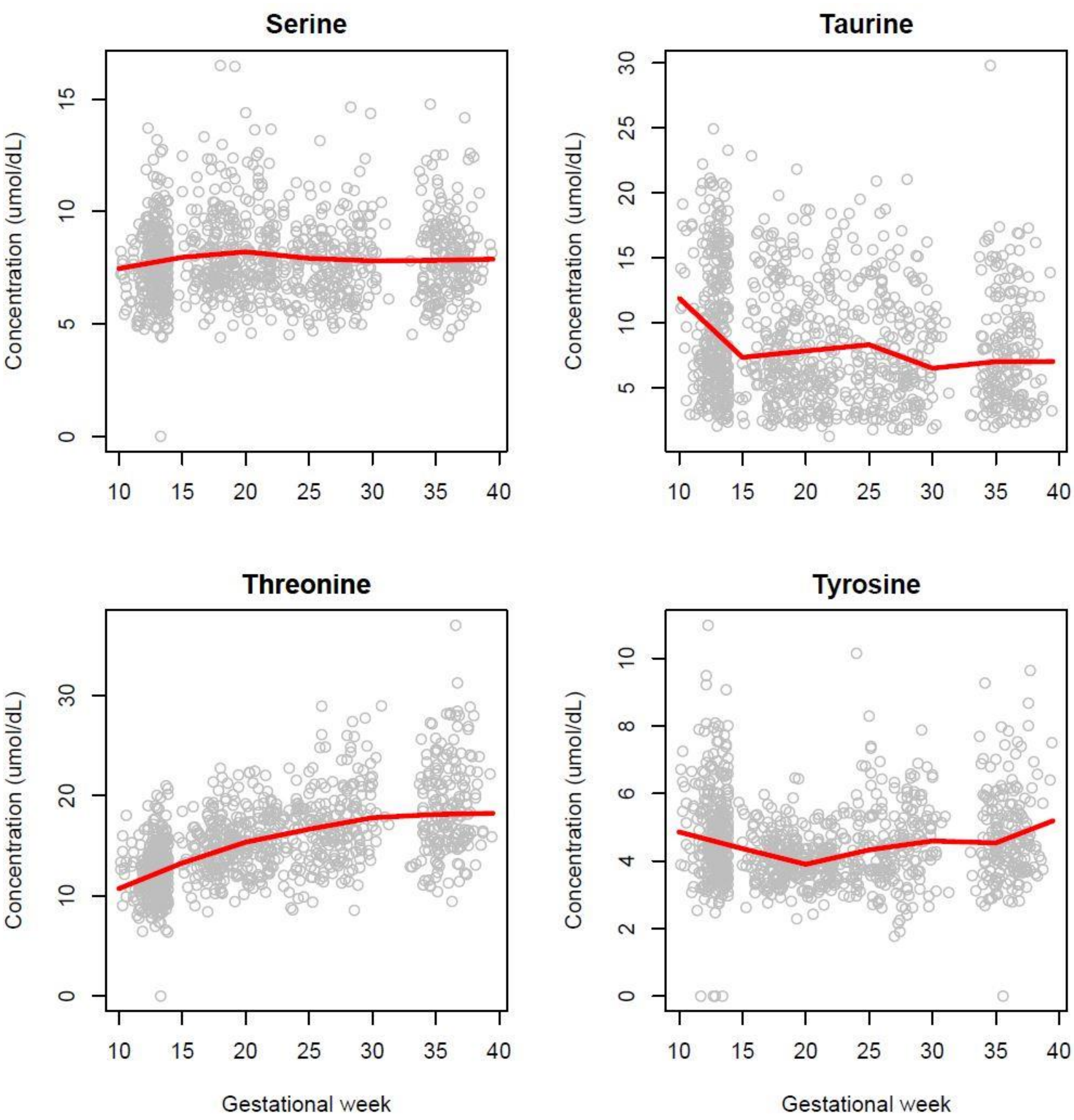
Serine
Taurine
Threonine
Tyrosine
Concentration (umol/dL)
Gestational week
10
15
20
25
30
35
40

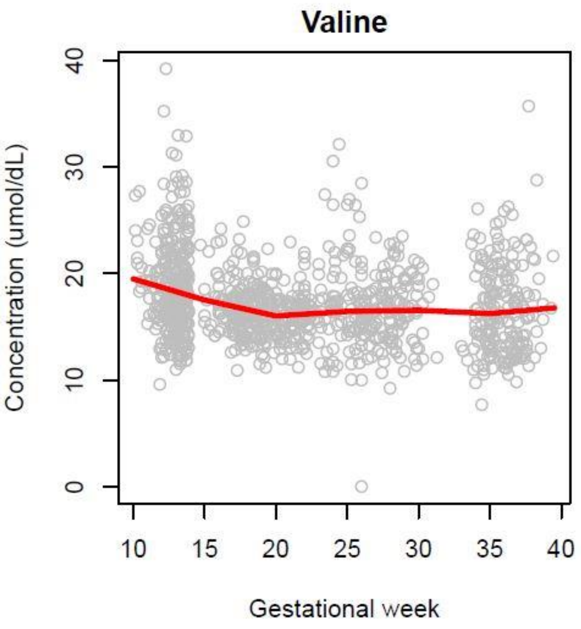
Valine
Concentration (umol/dL)
0
10
20
30
40
10
15
20
25
30
35
40
Gestational week
